# Secondary Metabolite Production Potential in a Microbiome of the Freshwater Sponge Spongilla lacustris

**DOI:** 10.1128/spectrum.04353-22

**Published:** 2023-02-02

**Authors:** Sophie Graffius, Jaime Felipe Guerrero Garzón, Martin Zehl, Petra Pjevac, Rasmus Kirkegaard, Mathias Flieder, Alexander Loy, Thomas Rattei, Andrew Ostrovsky, Sergey B. Zotchev

**Affiliations:** a Department of Pharmaceutical Sciences, Division of Pharmacognosy, University of Vienna, Vienna, Austria; b Department of Analytical Chemistry, Faculty of Chemistry, University of Vienna, Vienna, Austria; c Joint Microbiome Facility of the Medical University of Vienna and the University of Vienna, University of Vienna, Vienna, Austria; d Department of Microbiology and Ecosystem Science, Division of Microbial Ecology, Centre for Microbiology and Environmental Systems Science, University of Vienna, Vienna, Austria; e Department of Microbiology and Ecosystem Science, Division of Computational System Biology, Centre for Microbiology and Environmental Systems Science, University of Vienna, Vienna, Austria; f Doctoral School in Microbiology and Environmental Science, University of Vienna, Vienna, Austria; g Department of Palaeontology, Faculty of Earth Sciences, Geography and Astronomy, Geozentrum, University of Vienna, Vienna, Austria; h Department of Invertebrate Zoology, Faculty of Biology, Saint Petersburg State University, Saint Petersburg, Russia; Institut Ruder Boskovic

**Keywords:** bacterial isolates, biosynthetic gene clusters, freshwater sponge, metagenomics, microbiome, secondary metabolites

## Abstract

Marine and freshwater sponges harbor diverse communities of bacteria with vast potential to produce secondary metabolites that may play an important role in protecting the host from predators and infections. In this work, we initially used cultivation and metagenomics to investigate the microbial community of the freshwater sponge Spongilla lacustris collected in an Austrian lake. Representatives of 41 bacterial genera were isolated from the sponge sample and classified according to their 16S rRNA gene sequences. The genomes of 33 representative isolates and the 20 recovered metagenome-assembled genomes (MAGs) contained in total 306 secondary metabolite biosynthesis gene clusters (BGCs). Comparative 16S rRNA gene and genome analyses showed very little taxon overlap between the recovered isolates and the sponge community as revealed by cultivation-independent methods. Both culture-independent and -dependent analyses suggested high biosynthetic potential of the S. lacustris microbiome, which was confirmed experimentally even at the subspecies level for two *Streptomyces* isolates. To our knowledge, this is the most thorough description of the secondary metabolite production potential of a freshwater sponge microbiome to date.

**IMPORTANCE** A large body of research is dedicated to marine sponges, filter-feeding animals harboring rich bacterial microbiomes believed to play an important role in protecting the host from predators and infections. Freshwater sponges have received so far much less attention with respect to their microbiomes, members of which may produce bioactive secondary metabolites with potential to be developed into drugs to treat a variety of diseases. In this work, we investigated the potential of bacteria associated with the freshwater sponge *Spongilla lacustris* to biosynthesize diverse secondary metabolites. Using culture-dependent and -independent methods, we discovered over 300 biosynthetic gene clusters in sponge-associated bacteria and proved production of several compounds by selected isolates using genome mining. Our results illustrate the importance of a complex approach when dealing with microbiomes of multicellular organisms that may contain producers of medically important secondary metabolites.

## INTRODUCTION

Sponges (phylum Porifera) are among the most common and diverse sessile suspension feeders, inhabiting both marine and freshwater habitats. The phylum currently comprises more than 9,300 valid species (1), which are all holobionts hosting diverse bacterial and archaeal or algal-bacterial communities (2, 3). In contrast to marine sponges, whose microbial communities are well studied (reviewed in references 4 and 5), microbiomes of freshwater sponges have received much less attention, despite the fact that sponges are abundant elements of the freshwater benthic communities. Relatively few studies investigating freshwater sponges have been published (6–11), and only Clark et al. (12) reported on the secondary metabolite biosynthesis potential of two freshwater sponges’ microbiomes. Secondary metabolites are small molecules produced by a variety of living organisms, especially plants and microorganisms, via sometimes very complex biosynthetic pathways involving dozens of enzymes. These molecules appear not to be essential for proliferation of these organisms under laboratory conditions but may play an important role in the natural environment. For example, bacteria employ secondary metabolites to inhibit growth of competitors for nutritional sources (13), for communication between different species, genera, and even domains (14), and for scavenging metal ions (15). It has also been suggested that bacterial communities of sponges may biosynthesize defensive molecules to protect their hosts and host larvae from predators and infections (16–18). Metagenomics-based studies revealed a large diversity of secondary metabolite biosynthesis gene clusters (BGCs) harbored by bacterial sponge symbionts, but these reports focus mainly on BGCs that encode polyketide synthases (PKS) and/or nonribosomal peptide synthetases (NRPS) (19, 20). Identification of PKS and NRPS genes from environmental DNA is relatively straightforward, since these are perhaps the most abundant secondary metabolite biosynthesis genes and can be analyzed using selective PCR approaches.

In this study, we combined metagenomics and genomics of isolates for a more comprehensive analysis of the secondary metabolite biosynthesis potential of the microbiome of the freshwater sponge Spongilla lacustris (L., 1758), one of the most widespread sponge species found throughout temperate regions of the Northern Hemisphere. In Central Europe, it is the most common freshwater sponge species and one of the fastest-growing sponges, sometimes achieving up to a 1,000-fold increase in biomass during summer months (21, 22). While S. lacustris is a very effective biological filter that removes (and consumes) nearly all particulate matter in water, its growth mostly relies on photosynthesis-derived products supplied by the intracellular symbiotic algae (23, 24). To date, only one study on the bacterial community of *S. lacustris* has been published (7), describing the taxonomic diversity of sponge symbionts based on 16S rRNA gene amplicon analyses.

In the present work, we sequenced the 16S rRNA gene amplicon and the metagenome of *S. lacustris* and isolated representatives of 41 bacterial genera associated with this sponge. The 16S rRNA gene diversity analysis, however, clearly indicated that these genera do not represent a major part of the sponge microbiome. Analysis of both metagenome-assembled genomes (MAGs) recovered from the sponge metagenomes and genomes of representative isolates revealed the huge potential of the bacterial microbiota to synthesize diverse secondary metabolites. Moreover, using a genome mining approach, we activated expression of a BGC in one of the bacterial isolates and identified its products as congeners of the naphthoquinone-oxindole alkaloid coprisidin (25).

## RESULTS AND DISCUSSION

### Analyses of bacteria associated with *Spongilla lacustris*.

**FIG 1:**
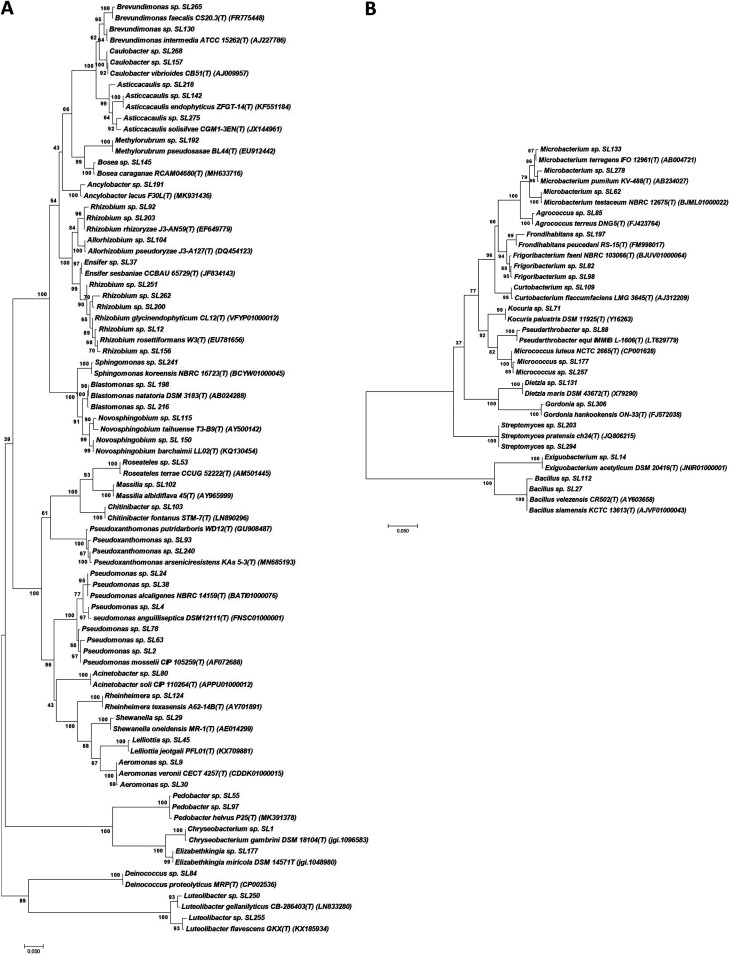
Phylogeny of bacteria isolated from *Spongilla lacustris* based on 16S rRNA gene fragment sequences. Most closely related type strains were included in the analyses. (A) Gram-negative bacterial isolates; (B) Gram-positive bacterial isolates. The evolutionary history was inferred by using the maximum likelihood method. The percentage next to each branch indicates the bootstrap value (1,000 resampling). Initial trees for the heuristic search were obtained automatically by applying neighbor-joining and BioNJ algorithms to a matrix of pairwise distances estimated using the maximum composite likelihood (MCL) approach and then selecting the topology with superior log likelihood value. The trees are drawn to scale, with branch lengths measured in the number of substitutions per site. Evolutionary analyses were conducted in MEGA 7.0 (50).

A sample of the freshwater sponge *S. lacustris* was collected from the Pichlinger See, Upper Austria (see Materials and Methods). The microbiome of the sponge was investigated using a combined approach of isolating bacteria and analyzing their genomes as well as analyzing 16S rRNA gene amplicons and metagenomes generated directly from sponge tissue DNA extracts. To isolate sponge-associated bacteria, sponge samples were homogenized to obtain a fine suspension. Incubation of serial dilutions of the sponge homogenate on nine different agar media over the period of up to 6 weeks at 22°C initially yielded 380 bacterial isolates, of which 183 either could not be cultivated as pure cultures or did not survive subculturing and storage. The 197 remaining isolates were identified using 16S rRNA gene sequencing, which, after phylogenetic analysis and dereplication, revealed representatives of 41 bacterial genera. Twenty-eight genera of Gram-negative bacteria and 13 genera of Gram-positive bacteria were represented by 49 and 19 isolates, respectively (Fig. 1A and B).

### Secondary metabolite biosynthesis potential of *Spongilla lacustris* bacterial isolates.

To investigate the secondary metabolite biosynthesis potential of bacteria isolated from *S. lacustris*, the genomes of 33 isolates representing 31 bacterial genera were sequenced and analyzed with antiSMASH 6.0 software (26) (Table 1). The isolates for genome sequencing were chosen based on their phylogeny. The genome sizes of the isolates varied widely, from 2.5 Mb for *Micrococcus* sp. SL257 to 7.8 Mb for *Streptomyces* sp. SL203 within Gram-positive species. *Ensifer* sp. SL37, *Roseateles* sp. SL47, *Rhizobium* sp. SL86, and *Massilia* sp. SL102 possessed the largest genomes among Gram-negative isolates: 8.2 Mb, 6.4 Mb, 6.0 Mb, and 7.4 Mb, respectively. Their genomes contained 10 or 11 detected BGCs, with dominating types being mostly ribosomally synthesized and posttranslationally modified peptides (RiPPs) and terpenes, except for *Rhizobium* sp. SL86, whose genome contained three BGCs attributed to the biosynthesis of unspecified homoserine lactones. We could not connect any of the BGCs in these isolates to known secondary metabolites when using the MiBIG database (27).

**TABLE 1:** Biosynthetic gene clusters detected in the genomes of *S. lacustris* bacterial isolates with antiSMASH 6.0 software (27)

Isolate	Genus	Genome, Mb	No. of biosynthetic gene clusters										
			PKS I	PKS II	PKS III	NRPS	PKS/nRPS	RiPP	Terpene	Siderophore	AHL	Other	Total
SL1	*Chryseobacterium*	5.1			1				1			2	4
SL4	*Pseudomonas*	4.4				1		3				3	7
SL14	*Exiguobacterium*	3.4							2	1			3
SL37	*Ensifer*	8.2	1					3	1	2	2	2	11
SL45	*Leliottia*	5.4				1		2				1	4
SL47	*Roseateles*	6.4				1	3	2	2			2	10
SL55	*Pedobacter*	4.4							1				1
SL62	*Microbacterium*	3.6			1	1		1	1			2	6
SL 71	*Kocuria*	3.4				1			1	1		1	4
SL75	*Microbacterium*	3.4			1	2		1	2			1	7
SL84	*Deinococcus*	4.0						3	1		1		5
SL85	*Agrococcus*	2.7			1	1			1			1	4
SL86	*Rhizobium*	6.0	1			1	1	1	1		3	3	11
SL 88	*Pseudoarthrobacter*	4.3			1	1				1		1	4
SL93	*Pseudoxanthomonas*	3.8					1	2				1	4
SL 95	*Pannonibacter*	5.0	1			1		2	1	1		1	7
SL 97	*Firgoribacterium*	3.7			1	1			1	1			4
SL 102	*Pseudoduganella*	7.4				2	1	2	2		1	3	11
SL 109	*Curtobacterium*	4.0			2	2		3	2			1	10
SL112	*Bacillus*	4.0	2		1	3	2	1	2			3	14
SL115	*Novosphingobium*	3.1			2				1				3
SL130	*Brevundimonas*	3.5						1					1
SL131	*Dietzia*	4.3	1			1			2	1		2	7
SL 142	*Asticcacaulis*	3.7				1		3					4
SL 161	*Caulobacter*	3.5					1					1	
SL191	*Angulomicrobium*	4.7	1			1		1	1		1	3	8
SL192	*Methylobacterium*	5.5	1			1			3	1	1	2	9
SL 203	*Streptomyces*	7.8	1	2	1	4	2	6	7	2		7	32
SL216	*Blastomonas*	3.9			1			4	2		1		8
SL250	*Luteolibacter*	4.9			1		1	1	2			1	6
SL257	*Micrococcus*	2.5							1	1		4	6
SL294	*Streptomyces*	7.0	1	1		3	2	6	6	2		4	25
SL306	*Gordonia*	5.4	1			5		2	3			3	14

In general, the previously suggested trend “larger genomes—more BGCs” (28) could be observed for both Gram-negative and Gram-positive bacterial isolates. However, there were some discrepancies regarding the ratio of the number of BGCs to the genome size. For example, *Curtobacterium* sp. SL109 and *Bacillus* sp. SL112 both had 4.0-Mb genomes and harbored 10 and 14 BGCs, respectively, while *Pedobacter* sp. SL55, with a 4.4-Mb genome, and *Brevundimonas* sp. SL130, with a 3.5-Mb genome, both harbored only 1 BGC of the terpene type each.

Several isolates, in particular *Ensifer* sp. SL37, *Deinococcus* sp. SL84, *Rhizobium* sp. SL86, *Pseudoduganella* sp. SL102, *Angulomicrobium* sp. SL191, *Methylobacterium* sp. SL192, and *Blastomonas* sp. SL216, harbor BGCs for the biosynthesis of *N*-acyl homoserine lactones (AHLs). These compounds are secondary metabolites commonly used as signaling molecules by Gram-negative bacteria in quorum sensing, regulating biofilm formation, virulence, motility, etc. (29). Moreover, AHLs have been implicated in communication between symbiotic bacteria and their hosts, such as plants and mammals, affecting the physiology of the latter (30, 31). It is thus plausible that the above-mentioned isolates could use AHLs to communicate with other bacteria as well as with their host, *S. lacustris*.

The bacteria with the highest secondary metabolite biosynthesis potential, as judged by the number of BGCs per genome, were the two *Streptomyces* isolates SL203 and SL294, which harbor 28 and 23 BGCs, respectively. *Bacillus* sp. SL112 and *Gordonia* sp. SL306 were the next BGC-rich isolates, both having 14 BGCs in their genomes. Eight out of 14 BGCs in the *Bacillus* sp. SL112 genome could be unequivocally linked to known secondary metabolites due to their high similarity (up to 98%) to known BGCs present in the genome of the plant growth-promoting bacterium Bacillus velezensis FZB42 (32). In particular, the SL112 isolate has the capacity to produce difficidin, bacillibactin, amylocyclicin, bacilysin, surfactin, macrolactin, bacillaene, and fengycin, though extensive experiments are required to confirm this. These compounds are polyketides, nonribosomally synthesized peptides, and hybrids of these two types of metabolites, which have a wide range of biological effects, including antibacterial, antifungal, and cytotoxic activities (33, 34). Strain SL112 might thus be involved in protecting its sponge host from microbial infections and predators and in modulating the host-associated bacterial community.

The genome of *Gordonia* sp. SL306 harbors the largest number of NRPS BGCs among all the genomes sequenced in this study. *Gordonia* is an actinobacterial genus for which very little is known about the secondary metabolite biosynthesis potential of its members. Although antimicrobial compounds such as actinomycins and mojavensin are produced by *Gordonia* (35), we could not identify any BGC in the genome of isolate SL306 that could be linked to known secondary metabolites. Since this actinobacterial genus is understudied, its representatives might be a source of novel bioactive natural products and shall be further investigated.

### Mining of the *Spongilla lacustris* metagenome for BGCs.

**FIG 2:**
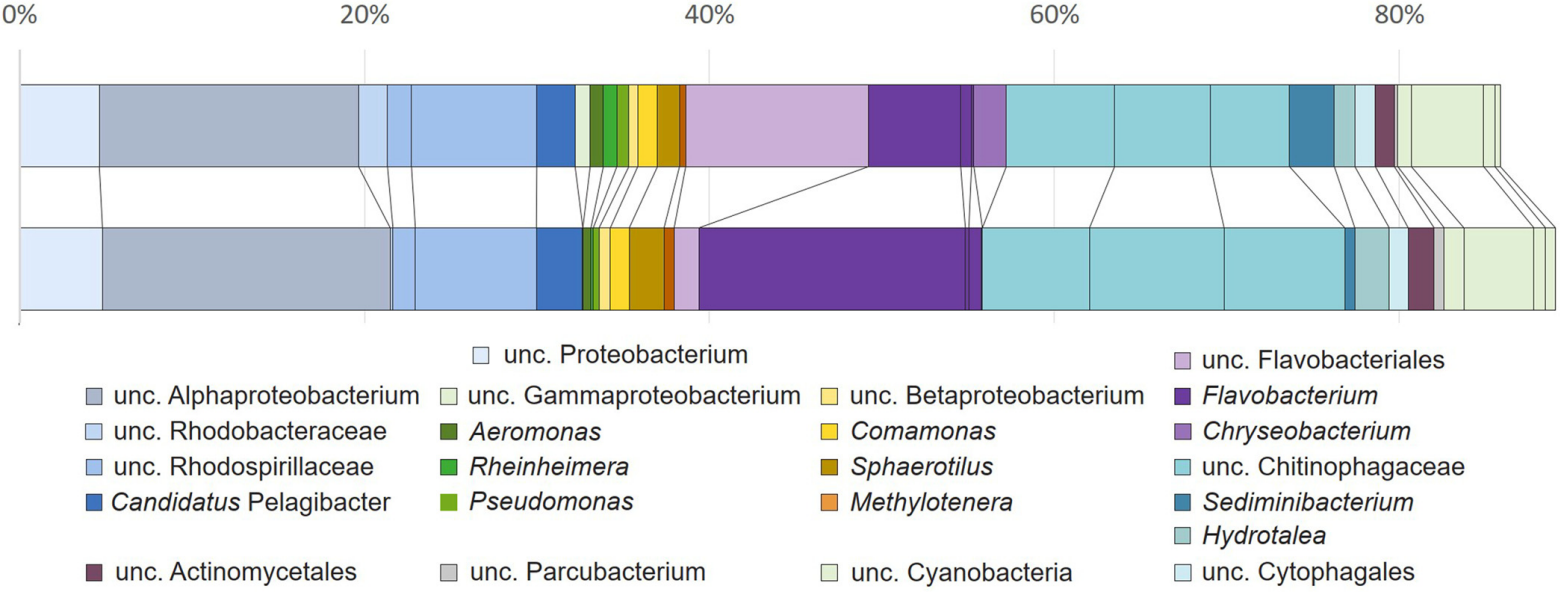
Cumulative relative 16S rRNA gene ASV abundance obtained from two *Spongilla lacustris* tissue DNA extracts. Relative abundances of obtained ASV were summarized at the genus level, where classification was possible, or at higher taxonomic levels (family, order, class, and phylum) where no further classification was possible. ASVs classified as bacteria, but with no classification at the phylum level are not depicted. ASVs related to the chloroplast of the dominant algal endosymbiont were removed before relative abundance calculations.

To evaluate the secondary metabolite biosynthesis potential of sponge-associated bacteria that could not be cultivated under the conditions used, the total DNA isolated from the sponge samples was used to generate 16S rRNA gene amplicon libraries and a metagenome library, both of which were sequenced. The 16S rRNA gene sequencing revealed a large degree of phylogenetic novelty in the *S. lacustris* microbiome, as many abundant amplicon sequence variants (ASVs) could not be classified at the genus level and were predominantly related to various uncultured and unclassified clades affiliated with *Bacteroides*, *Alphaproteobacteria*, *Gammaproteobacteria*, and *Betaproteobacteria* (Fig. 2).

**FIG 3:**
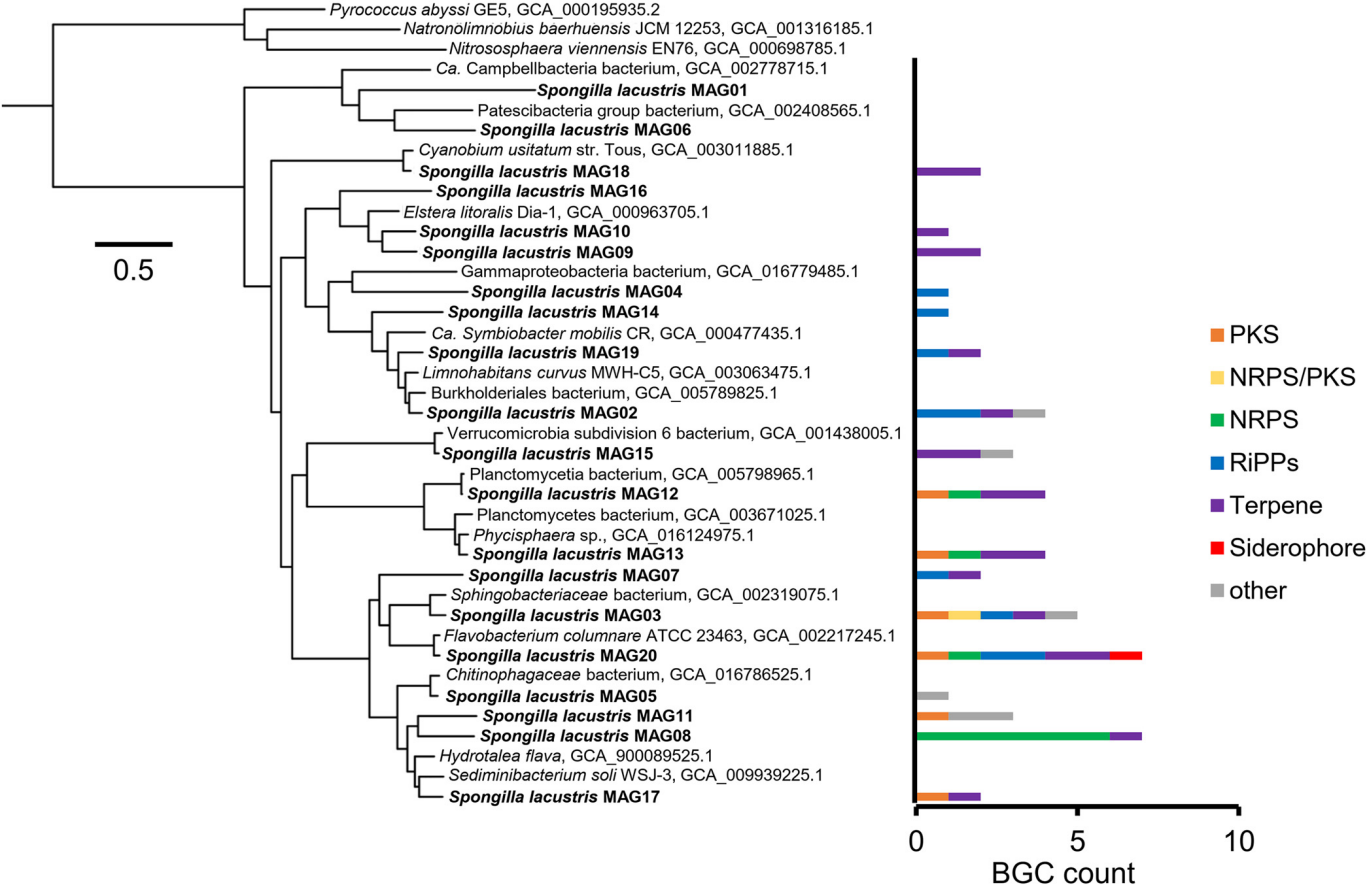
Maximum likelihood tree of concatenated protein sequences of single marker genes retrieved with CheckM from MAGs and reference genomes/MAGs. The number and types of biosynthetic gene clusters (BGCs) that are encoded on each MAG obtained in this study are shown. Pyrococcus abyssi GE5, Nitrososphaera viennensis EN76, and Natronolimnobius baerhuensis JCM 12253 were used as an outgroup. The scale bar represents 0.5 substitution per site. Accession numbers of genomes/MAGs are given next to the organism’s name where applicable.

Metagenome binning resulted in 20 medium- to high-quality metagenome-assembled genomes (MAGs) with at least 50% completeness and less than 10% contamination based on CheckM (see Table S1 in the supplemental material). The sizes of the MAGs varied between 0.48 and 4.98 Mb, and with an average size of 2.5 Mb, the MAGs were rather small. According to the classification with the Genome Taxonomy Database Toolkit (GTDB-Tk), the MAGs belong to six phyla representing *Cyanobacteria*, *Proteobacteria*, *Bacteroidota*, *Verrucomicrobiota*, *Planctomycetota*, and *Patescibacteria* (Table S1), which reflects the diversity and composition of the community based on 16S rRNA gene amplicon sequencing. Based on the MAGs’ placement in the GTDB phylogenomic tree, no sponge-related taxa were observed within their respective clade. Most of the closest related MAGs within the GTDB were from metagenomes of freshwater origin but also a hot spring and a hypersaline lake (Table S1). Within their clades, MAGs and genomes additionally belonged to marine water metagenomes or isolates but also intracellular pathogens of shrimps, symbionts of *Paramecium*, or isolates from diseased fish (data not shown). In total, 51 BGCs were identified in all MAGs, but three MAGs had no BGCs detected (Table 2 and Fig. 3). Two *Bacteroidota* MAGs had the highest number of BGCs, with seven each. Most detected BGCs encoded the secondary metabolite class of terpenes, followed by RiPPs and NRPS, yet one *Bacteroidota* MAG alone already accounted for most of those NRPS.

**TABLE 2:** Biosynthetic gene clusters detected in the MAGs of uncultured bacteria associated with *S. lacustris* using antiSMASH 6.0 software (27)^*a*^

MAG	Phylum_gtdb	No. of biosynthetic gene clusters							
		PKS III	NRPS/pKS	NRPS	RiPPs	Terpene	Siderophore	Other	Total
MAG01	*Patescibacteria*								0
MAG02	*Proteobacteria*				2	1		1	4
MAG03	*Bacteroidota*	1	1		1	1		1	5
MAG04	*Proteobacteria*				1				1
MAG05	*Bacteroidota*							1	1
MAG06	*Patescibacteria*								0
MAG07	*Bacteroidota*				1	1			2
MAG08	*Bacteroidota*			6		1			7
MAG09	*Proteobacteria*					2			2
MAG10	*Proteobacteria*					1			1
MAG11	*Bacteroidota*	1						2	3
MAG12	*Planctomycetota*	1		1		2			4
MAG13	*Planctomycetota*	1		1		2			4
MAG14	*Proteobacteria*				1				1
MAG15	*Verrucomicrobiota*					2		1	3
MAG16	*Proteobacteria*								0
MAG17	*Bacteroidota*	1				1			2
MAG18	*Cyanobacteria*					2			2
MAG19	*Proteobacteria*				1	1			2
MAG20	*Bacteroidota*	1		1	2	2	1		7

a PKS I and PKS II clusters were not detected in the MAGs.

The phylogenetic distance between the obtained MAGs and the isolate genomes was too high for calculating average nucleotide identities (ANI). This suggests that despite using a wide range of isolation media, we could not cultivate those bacteria that, according to the metagenome and 16S rRNA gene amplicon sequencing analysis, represent the most abundant species associated with the sponge. The pure isolates may thus represent either low-abundance members of the bacterial community or bacteria that derive from surrounding water temporarily adhered to or filtered by the sponge.

### Secondary metabolites produced by two closely related *Streptomyces* isolates.

*Streptomyces* isolates SL203 and SL294 have the highest number of BGCs and are also very closely related (Fig. 1B), with more than 99.9% identity of their 16S rRNA gene sequences. Nevertheless, the isolates were distinct in terms of morphology. While isolate SL203 displayed a rather characteristic morphology on soy flour medium (SFM), forming round soft colonies with gray spores, SL294 did not sporulate on the same medium and formed yellow-pigmented hard colonies (Fig. S1). Furthermore, differences were noticed regarding antibiotic resistance. While SL294 was sensitive to hygromycin, SL203 was resistant against this antibiotic (data not shown). The genomes of these isolates differed considerably in size, with 7.85 Mb for SL203 and 6.98 Mb for SL294. Despite these differences, the ANI value for the isolates determined with EzBioCloud ANI calculator (https://www.ezbiocloud.net/tools/ani) was 99.95%, clearly suggesting that they belong to the same species. The difference in genome sizes correlated with the BGC content. The two isolates shared 23 BGCs, but SL203 harbored 5 additional BGCs that were not present in SL294.

Given that several of those BGCs were uncharacterized and therefore could be involved in the biosynthesis of novel bioactive compounds, isolates SL203 and SL294 were subjected to genome mining. First, a gene transfer system was established for both and showed a clear difference between them, since the number of SL203 transconjugants that could be obtained with the pSET152 vector was almost 2 orders of magnitude greater than that for SL294. We were particularly interested in a type II PKS BGC identified in the SL203 and SL294 isolates (BGCs 3.9 and 4.12 in Tables S3 and S4, respectively), because we could not directly connect them to a known secondary metabolite. This BGC spans ca. 40 kb and contains core genes for the biosynthesis of a type II polyketide along with the genes encoding tailoring enzymes and regulators. A gene (ctg3_1243) encoding a putative streptomyces antibiotic regulatory protein (SARP)-type transcriptional regulator was identified within the BGC and cloned under the control of the strong promoter ermE*p in the newly constructed pSET152-based vector.

Methanolic extracts of cultures from isolates SL203 and SL294 bearing pSET152_ermE*p empty vector and SARP-overexpressing plasmid were analyzed by liquid chromatography-mass spectrometry (LC-MS), and known secondary metabolites were tentatively identified by a tandem mass spectrometry (MS/MS) spectrum library and compound database search (Fig. S2 to S4). It should be noted that while LC-MS-based metabolomics is very sensitive, it provides only limited structural information compared to nuclear magnetic resonance (NMR) spectroscopy and X-ray crystallography, particularly regarding the stereochemistry, and thus largely relies on previously published data.

The dereplication of the common *Streptomyces* natural products nocardamine (36), dehydroxynocardamine (37), coelichelin (38), and ectoine (39), all matching the antiSMASH predictions in both strains (Tables S3 and S4), was straightforward based on comparison to published MS/MS data (Fig. S5 to S8). In addition, a coproporphyrin (40) was detected in both strains in SM17 medium (Fig. S9). Based on the accurate mass and isotopic pattern, a large number of isomers matching sceliphrolactam and tripartilactam were observed (Fig. S10 and S11). No reference MS/MS spectra were available for these compounds, but their identification was supported by comparison of the assigned BGC (Tables S3 and S4), the UV spectra, and the complex chromatogram resulting from the spontaneous conversion of sceliphrolactam to tripartilactam to previously published data (41).

For the majority of known natural products there are no MS/MS library spectra available and they are not yet connected to their BGCs. These compounds could still be tentatively identified by LC-MS with careful data interpretation and literature search, but this workflow is very time-consuming and inefficient due to the high chemical complexity of culture medium extracts. In the case of isolates SL203 and SL294, we could exploit the fact that the former possesses all the predicted BGCs of the latter while harboring five additional ones. Thus, we hypothesized that by comparing the extracts from the two isolates we would identify the products of the additional BGCs in SL203. Indeed, an abundantly produced compound present exclusively in the extracts of *Streptomyces* sp. SL203 was identified as recently described detoxin S1 (Fig. S12 and S13) and matched to the NRPS-type BGC 3.2 (Table S3). This member of the detoxin/rimosamide family was discovered in *Streptomyces* sp. NRRL S-325 by a computational workflow and MS/MS data interpretation, but a full structure elucidation is still lacking (42).

**FIG 4:**
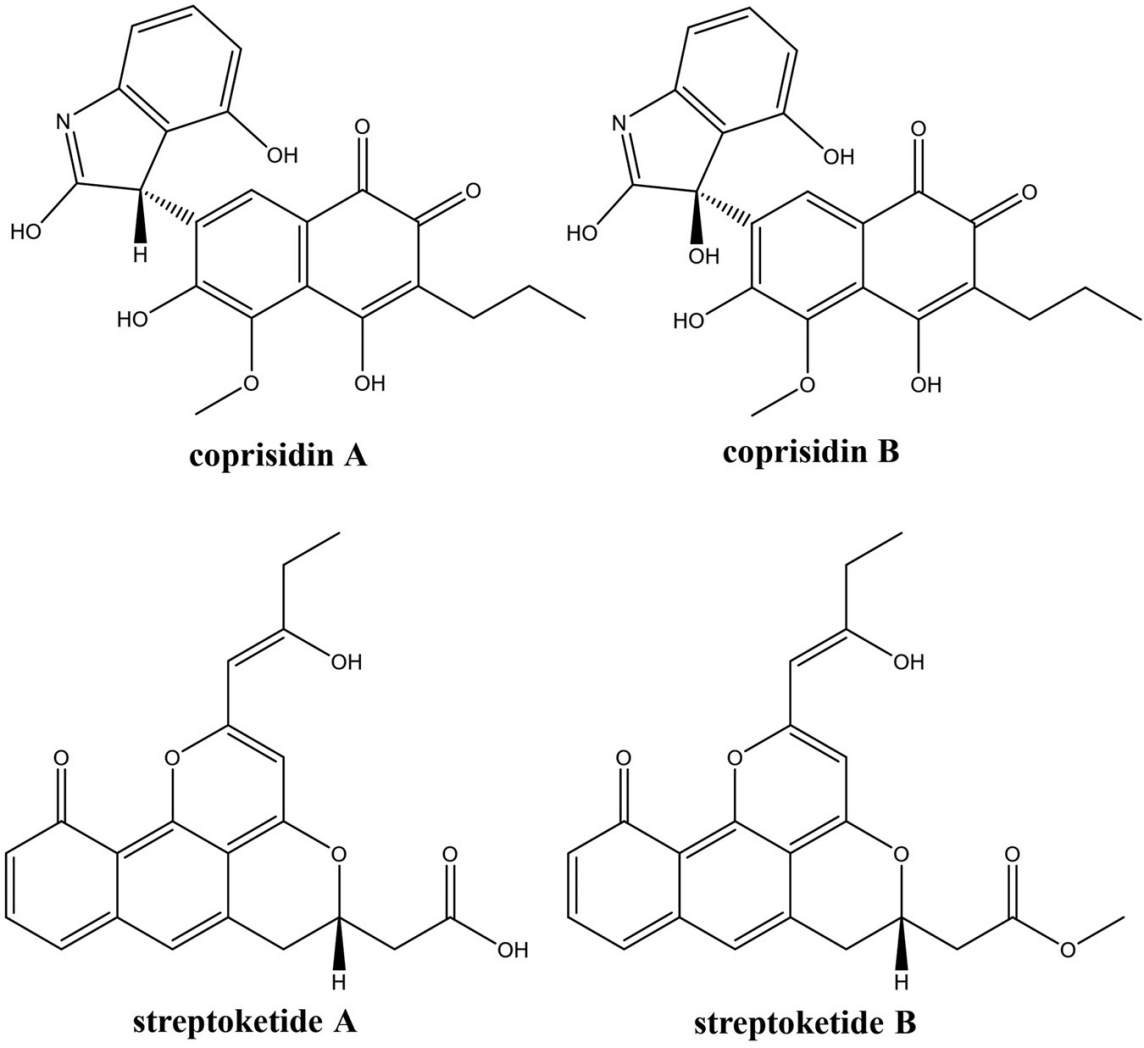
Chemical structures of coprisidins (25) and streptoketides (44).

The constitutive overexpression of the SARP regulator in both SL203 and SL294 led to the specific upregulation of the production of several metabolites in both strains (Fig. S3 and S4), two of which were tentatively identified as the naphthoquinone-oxindole alkaloids coprisidins A and B (25) by LC-MS (Fig. S14 and S15). For confirmation, coprisidin A was isolated by preparative high-performance liquid chromatography (HPLC) and its structure verified by comparison of the obtained ^1^H and ^13^C NMR spectra with literature data (Table S5 and Fig. S16 and S17). The biosynthetic gene cluster for coprisidins was recently identified in *Streptomyces* sp. SNU607, isolated from the dung beetle Copris tripartitus (43). The gene composition of the coprisidin BGC fits perfectly to those of BGCs 3.9 and 4.12 in the genomes of isolates SL203 and SL294, respectively. In a previous study, a BGC almost identical in gene composition from *Streptomyces* sp. Tü 6314 has been cloned and heterologously expressed (44), leading to the production of streptoketides. Coprisidins and streptoketides are structurally dissimilar (Fig. 4), with only the former compounds bearing an oxindol moiety. We compared the genes in the three BGCs in order to potentially identify the ones responsible for the appearance of the oxindol moiety in the coprisidins (Table 3). Despite some differences in the annotations (original annotation from references 43 and 44 is retained in Table 3), the products of the genes in shaded cells of Table 3 are 90% to 99% identical, strongly suggesting identical functions. The most probable reason for production of streptoketides instead of coprisidins upon heterologous expression of the cluster in Streptomyces coelicolor is the disbalanced transcription of certain genes in this heterologous host, in particular those responsible for installation of the oxindole moiety. It is worth noting that while streptoketides were shown to have antiviral activity, coprisidin A inhibited the action of Na^+^/K^+^-ATPase, and coprisidin B activated NAD(P)H:quinone oxidoreductase 1 (25). Hence, modulating expression of specific genes in the coprisidin BGC by environmental factors may lead to production of analogues and precursors with variable biological activities, representing a phenotypic plasticity that may be beneficial for the sponge in terms of defense against predators and infections.

**TABLE 3:** Comparison of the BGCs for streptoketides and coprisidins^*a*^

Streptoketides, *Streptomyces* sp. Tü 6314		Coprisidins, *Streptomyces* sp. SNU 607		Coprisidins, *Streptomyces* sp. SL203	
Gene	Annotation	Gene	Annotation	Gene	Annotation
		*orf32*	AP-4-A phosphorylase	ctg3_1252	HIT domain protein
*L2*	Hypothetical protein	*orf33*	Hypothetical protein	ctg3_1251	DUF6059 domain protein
				ctg3_1250	Hypothetical protein
*L1*	NmrA/HSCARG family protein	*copQ*	NAD-dependent ketoductase/epimerase	ctg3_1249	NAD-dependent epimerase/dehydratase
*skt1*	Acyl carrier protein	*copA*	Acyl carrier protein	ctg3_1248	Acyl carrier protein
*skt2*	Ketosynthase β (chain length factor)	*copB*	Ketosynthase β (chain length factor)	ctg3_1247	Ketosynthase β (chain length factor)
*skt3*	Ketosynthase α	*copC*	Ketosynthase α	ctg3_1246	Ketosynthase α
*skt4*	MarR family transcriptional regulator	*copR1*	MarR family transcriptional regulator	ctg3_1245	MarR family transcriptional regulator
*skt5*	Cyclase	*copD*	Dehydratase/cyclase	ctg3_1244	SnoaL-like domain protein
*skt6*	Regulator	*copR2*	SARP family regulator	ctg3_1243	SARP family regulator
*skt7*	GMC family oxidoreductase	*copS*	Cholesterol oxidase	ctg3_1242	GMC oxidoreductase
*skt8*	SAM-dependent methyltransferase	*copT*	*O*-Methyltransferase	ctg3_1241	*O*-Methyltransferase
*skt9*	Ketoacyl reductase	*copE*	Ketoreductase	ctg3_1240	Short-chain dehydrogenase
*skt10*	Aromatase/cyclase	*copF*	Cyclase/dehydrase	ctg3_1239	Polyketide cyclase/dehydrase
*skt11*	CoA ligase	*copG*	Acyl-CoA ligase	ctg3_1238	Acyl-CoA ligase
*skt12*	SDR family oxidoreductase	*copH*	3-Oxoacyl-ACP reductase	ctg3_1237	Enoyl-ACP reductase
				ctg3_1236	Short-chain dehydrogenase
*skt13*	Reductase	*copI*	Enoyl-ACP reductase III	ctg3_1235	Enoyl-ACP reductase
*skt14*	Monooxygenase	*copJ*	FAD-binding monooxygenase	ctg3_1234	FAD-dependent oxidoreductase
*skt15*	Cyclase	*copK*	Cyclase	ctg3_1233	Cyclase
*skt16*	Aldo/keto reductase	*copL*	Aldo/ketoreductase	ctg3_1232	Aldo/ketoreductase
*skt17*	3-Oxoacyl-ACP synthase	*copM*	Ketosynthase III	ctg3_1231	3-Oxoacyl-ACP synthase III
				ctg3_1230	Hypothetical protein
*skt18*	Acyltransferase domain-containing protein	*copN*	Acyltransferase	ctg3_1229	Acyltransferase
*skt19*	Methylmalonyl-CoA carboxyltransferase	*copO*	Acyl-CoA carboxyl transferase	ctg3_1228	Carboxyl transferase
				ctg3_1227	Phosphoethanolamine transferase domain protein
*R1*	Membrane protein	*copP*	Membrane protein		
*R2*	Elongation factor G	*orf55*	Translation elongation factor G-related protein	ctg3_1226	Elongation factor G
		*orf56*	CDP-diacylglycerol-glycerol-3-phosphate 3-phosphatidyltransferase	ctg3_1225	CDP-alcohol phosphatidyltransferase

a Shading indicates that the products of the genes are 90% to 99% identical, which strongly suggests identical functions. CoA, coenzyme A; ACP, acyl carrier protein.

Overexpression of the SARP regulator from the coprisidin BGC had also some untargeted effects. In SL294, the production of nonactin and related polyether ionophores (45) was triggered by the overexpression of the SARP regulator (Fig. S18 and S19). Another compound with the sum formula C_25_H_31_O_5_N was found to be biosynthesized in much larger amounts in the SARP-overexpressing strains, but mainly in SL294 and only in an ~1,000-fold-lower concentration in SL203. This metabolite was not identified by the LC-MS workflow since it was not included in the GNPS library, the Dictionary of Natural Products (https://dnp.chemnetbase.com/chemical/ChemicalSearch.xhtml?dswid=1396), or the CAS registry. Structure elucidation after isolation by preparative HPLC and extensive one-dimensional (1D) and 2D NMR spectroscopic experiments (Table S6 and Fig. S20 to S26), with the help of The Natural Products Atlas (46), finally showed this compound to be identical to saccharoquinoline, a cytotoxic alkaloidal meroterpenoid isolated from the marine-derived actinomycete *Saccharomonospora* sp. CNQ-490 (47). However, analyses of the genome of the latter bacterium with antiSMASH and comparison of the results to those for isolate SL294 did not allow straightforward identification of the saccharoquinoline BGC.

Our results for these two strains of the same *Streptomyces* species strongly suggest that dereplication of streptomycete isolates, i.e., selection for downstream secondary metabolite discovery, cannot be done solely on the basis of their 16S rRNA gene sequence-inferred taxonomy (48). Apparently, streptomycetes are capable of reshuffling their genomes either by acquisition of BGCs from other bacteria or by losing parts of them, thus modifying their genomes and equipping their cells with an ability to produce chemically diverse secondary metabolites that may play an important role in their environmental adaptation.

## MATERIALS AND METHODS

### Sampling and processing of the sponge sample.

The entire (i.e., nonfragmented) *Spongilla lacustris* sponge was collected at a 4.5-m depth by SCUBA diving on 5 September 2019 in Pichlinger See, Upper Austria (48°14′24″N, 14°22′53″E). The sponge grew on the silty patch surrounded by macrophytes. The sponge was transported to the lab in a sterile container in water from the lake, dissected upon arrival, and rinsed in sterile water. Approximately 4 g of the sponge sample was subjected to homogenization in 20% sterile glycerol (40 s, 4,500 rpm) using a Precellys 24 tissue homogenizer (Bertin Instruments, France). Part of the homogenized sample was used to make serial dilutions for bacterial isolation (see below); the rest was stored at −80°C and later used for isolation of DNA for 16S rRNA gene amplicon and metagenome-based community analysis.

### Isolation of bacteria.

Nine different media were used for isolation of bacteria: ISP2 (Difco), ISP4 (Difco), Actino isolation agar (Sigma-Aldrich), YIM11 (glycerol [5 g/L], arginine [0.5 g/L], glucose [1 g/L], K_2_HPO_4_ [0.3 g/L], MgSO_4_·7H_2_O [0.2 g/L], NaCl [0.3 g/L], agar [20 g/L] [pH 7.2], supplemented with 0.5 mg each of thiamine hydrochloride [vitamin B_1_], riboflavin, niacin, pyridoxine, calcium pantothenate, inositol, *p*-aminobenzoic acid, and 0.25 mg of biotin), Czapek’s Dox (Sigma-Aldrich), Trypticase soy agar (TSA; Sigma-Aldrich), King’s B (Sigma-Aldrich), starch casein agar (soluble starch [10 g/L], casein [vitamin free; 0.3 g/L], KNO_3_ [2 g/L], MgSO_4_·7H_2_O [0.05 g/L], K_2_HPO_4_ [2 g/L], NaCl [2 g/L], CaCO_3_ [0.02 g/L], FeSO_4_·7H_2_O [0.01 g/L], agar [18 g/L] [pH 7.0]), and sodium propionate agar (sodium propionate [1 g/L], l-asparagine [0.2 g/L], KH_2_PO_4_ [0.9 g/L], K_2_HPO_4_ [0.6 g/L], MgSO_4_·7H_2_O [0.1 g/L], CaCl_2_·2H_2_O [0.2 g/L], agar [18 g/L] [pH 7.2]), containing either nystatin (50 μg/mL) plus cycloheximide (50 μg/mL) or nystatin (50 μg/mL) plus cycloheximide (50 μg/mL) plus nalidixic acid (30 μg/mL). The homogenized sponge was used for serial dilution in H_2_O (1:10) until dilution –5; dilutions were spread onto the isolation plates. The plates were stored in the dark at room temperature or at 28°C, depending on the growth. Colonies were picked with a sterile toothpick and streaked on agar plates containing the above-mentioned supplementation of antibiotics. If there was growth, liquid cultures (3 mL) in LB/Trypticase soy broth (TSB)/TSB diluted (1:5) or liquid actinomycete isolation medium were prepared and incubated at 200 rpm and 28°C. If they did not grow in liquid cultures, agar plates were inoculated with 1 isolate/plate to gain enough biomass for DNA isolation. (e.g., if the colony appeared on TSA, TSA was also used to gain enough biomass). Half of the cultures (plate or liquid culture) was used for DNA isolation, and the other half was used to prepare 20% glycerol stocks, which were stored at −80°C.

### DNA extraction from isolates, isolate 16S rRNA gene sequencing, and taxonomic classification.

Genomic DNA was extracted by using the kit NucleoSpin microbial DNA for DNA, RNA, and protein purification by Macherey & Nagel, following their protocol for extracting microbial DNA. Cell disruption, as demanded in the protocol, was carried out on the Precellys 24 tissue homogenizer with 4,000 rpm for 1 cycle of 60 s. Amplification of ca. 1,400-bp 16S rRNA gene fragments was done using primers 27F and 1492R as described previously (49). Sequencing of the PCR fragments was accomplished at Eurofins (Germany). The obtained sequences were assembled with Clone Manager 9 software. The assembled sequences were analyzed with RDP and EzBiocloud databases. Phylogenetic analyses of 16S rRNA genes were performed with MEGA 7.0 software (50), and analysis of binned genomes was done with IQ-TREE software (51). The sequences were deposited to GenBank under accession numbers OP593123 to OP593300.

For isolation of genomic DNA intended for genome sequencing, 25 mL of medium (TSB, liquid R2A, or nutrient medium) was inoculated with 200 μL of glycerol stock or 0.5 to 2 mL of a 10-mL preculture inoculated with 100 μL of glycerol stock and incubated until dense cultures were obtained at 200 rpm and 25 to 28°C. Pellets from these cultures were used to isolate genomic DNA with the Wizard DNA isolation kit by Promega, following their protocol for isolation of DNA from bacteria but using a 10-fold volume of reagents and some modifications including incubation on ice for 10 to 20 min and an additional washing step with phenol-chloroform-isoamyl alcohol (25:24:1, vol/vol/vol; Roti phenol; Roth) before rehydration. Some of the genomic DNAs were isolated with the Power Soil Pro kit (Qiagen, Germany) following the manufacturer’s protocol at the Joint Microbiome Facility (Medical University of Vienna and University of Vienna) from the cell pellets prepared from 10- to 15-mL cultures of TSB, TSB diluted with water (1:1), liquid R2A, or nutrient medium incubated at 25 to 28°C and 200 rpm until dense growth (optical density at 600 nm [OD_600_] > 2) was observed.

### Genome/metagenome sequencing and analyses.

Genomic DNAs extracted from pure cultures of bacterial isolates were sequenced using the Illumina and Oxford Nanopore platforms. The DNA was prepared for Nanopore sequencing using the rapid barcoding sequencing kit (SQK-RBK004; Oxford Nanopore Technologies) following the manufacturer’s protocol. The DNA was sequenced on a MinION Mk1b (Oxford Nanopore Technologies) on a R9.4.1 flow cell (FLO-MIN106D; Oxford Nanopore Technologies) using Minknow (v. 20.10.3; Oxford Nanopore Technologies). For Illumina sequencing, DNA libraries were prepared with the NEBNext Ultra II FS DNA library prep kit (New England Biolabs) sequenced on the Illumina MiSeq platform (v3 chemistry, 2 × 300 cycles). Nanopore reads were basecalled using Guppy (v. 6.0.6) using super accuracy mode, while Illumina reads were quality trimmed using cutadapt (v. 3.1) (52) before further processing. The Nanopore reads were assembled using flye (v. 2.9-b1768) (53) with “–nano-hq” and polished three times with minimap2 (v. 2.17) (54) and racon (v. 1.4.3) (55), followed by two rounds of polishing with medaka (v. 1.4.4; https://github.com/nanoporetech/medaka). The genomes were further polished with the Illumina data using minimap2 (v. 2.17) (54) and racon (v. 1.4.3) (55). Reads were mapped to the assemblies using minimap2 (v. 2.17) (54), read mappings were converted using samtools (v. 1.12) (56), and read coverage was calculated using metabat2 (v. 2.15) (57). The quality of the genomes was checked using QUAST (v. 5.0.2) (58), CheckM (v. 1.1.1) (59), and genomes were classified using GTDB-Tk (v. 2.1.0) (60).

Metagenomic DNA was isolated from the sponge sample using the Power Soil Pro DNA extraction kit (Qiagen, Germany), libraries were prepared with the NEBNext Ultra II FS DNA library prep kit (New England Biolabs) and sequenced on the Illumina NovaSeq6000 platform (SP flow cell, 2 × 150 cycles).

16S rRNA gene sequences were reconstructed from quality-filtered metagenomic read data using phyloflash (v. 3.3) (61). The abundance table was loaded into R (v. 4.0.3) and visualized using the ampvis2 R package (v. 2.6.8) (62). The metagenomic sequences were assembled using MetaSpades 3.13.0 (63) using default parameters. The assembly was binned into metagenome-assembled genomes (MAGs) using MetaBat 2 (v. 2.15) (57) and MaxBin 2 (64). DasTool (v. 1.1.0) (65) was used to aggregate the MAGs from the two binning approaches and then dereplicated using drep (v. 3.0.0) (66) with an average nucleotide identity (ANI) cutoff of 95%. MAGs with at least 50% completeness and less than 10% contamination were retrieved. For taxonomic classification of the MAGs, GTDB-Tk (v. 0.3.3) (60) was used, and CheckM (v. 1.1.3) (59) was used for estimations of completeness and contamination of the MAGs as well as for retrieving single-copy marker genes for treeing. The GTDB phylogenomic tree was visualized using ARB (v. 7.0) (67). A phylogenomic maximum likelihood tree was calculated using the IQ-TREE (51) web server with substitution model AUTO and ultrafast 1,000× bootstrapping. The tree was visualized using iTOL (68). MAGs were analyzed for BGCs using the online tool antiSMASH 6.0 (26).

### 16S rRNA gene amplicon sequencing and analysis.

Hypervariable region V4 of bacterial and archaeal 16S rRNA genes was amplified using the primer pair 515F (69) and 806R (70) amplified in a 2-step PCR approach and sequenced at the Joint Microbiome Facility of the Medical University of Vienna and the University of Vienna (project identifiers [IDs] JMF-1909-01) as described in detail by Pjevac et al. (71). Amplicon pools were extracted from raw sequencing data with the FASTQ workflow under default settings (BaseSpace; Illumina); residual PhiX contamination was removed with BBDuk (B. Bushnell, https://sourceforge.net/projects/bbmap) and amplicons demultiplexed, allowing one mismatch to barcode and two to primer/linker sequences using the python package demultiplex (J. F. J. Laros, https://github.com/jfjlaros/demultiplex). The DADA2 R package v. 1.20.0 (R 4.1.1) (72, 73) was used to infer amplicon sequence variants (ASVs), which were classified based on SILVA taxonomy (reference no. 99, release 138.1) using the SINA version 1.6.1 (74) classifier. ASVs classified as eukaryotes, mitochondria, or chloroplasts, as well as singleton and doubleton ASVs, were removed prior to downstream analysis.

### Bacterial strains and growth conditions used for genome mining.

Escherichia coli DH5α was used to maintain plasmids. E. coli ET12567/pUZ8002 was used to perform conjugations of plasmids into streptomycetes. All E. coli strains were cultivated in liquid and solid LB medium at 37°C. *Streptomyces* spp. were grown at 28°C in soy flour medium (SFM) (75) followed by preparation of spore suspensions in 20% glycerol. Appropriate antibiotics were supplemented as necessary (50 μg/mL of apramycin, 50 μg/mL of nalidixic acid, 25 μg/mL of kanamycin, and 25 μg/mL of chloramphenicol).

### Construction of the pSET152_ermE*p plasmid and activation of the coprisidin biosynthetic gene cluster.

The strong ermE*p promoter was amplified by PCR from plasmid pSOK806 (76); the primers ermEp_Fw and ermEp_Rv were used (see Table S2 in the supplemental material). Primer ermEp_Fw incorporated the XbaI restriction site to the generated DNA fragment (433 bp) that was digested with XbaI and BamHI enzymes. The resulting DNA fragment (304 bp) was ligated to the digested pSET152 plasmid to generate the pSET152_ ermE*p plasmid. A putative transcriptional activator gene (ctg3_1243) that belongs to the SARP family was identified in cluster 3.9. A DNA fragment (937 bp) representing the ctg3_1243 gene was amplified by PCR from genomic DNA (gDNA) of *Streptomyces* sp. SL203; the primers ctg3_1243_Fw and ctg3_1243_Rv were used (Table S2). These primers included the restriction sites NotI and EcoRI, respectively, to allow proper ligation with the pSET152_ermE*p-digested fragment. The resulting construct was introduced into *Streptomyces* sp. SL203 and *Streptomyces* sp. SL294 via conjugation from E. coli ET12567/pUZ8002 (77). Apramycin (50 μg/mL) and nalidixic acid (30 μg/mL) were used for selection of recombinant *Streptomyces* strains. Plasmid pSET152_ermE*p was used as a control for conjugation and as negative control in further analysis of secondary metabolite production.

### Secondary metabolite identification and characterization.

Ten milliliters of YEME medium (yeast extract [3 g/L], peptone [5 g/L], malt extract [3 g/L], glucose [10 g/L], sucrose [340 g/L]) containing apramycin (50 μg/mL) was inoculated with 50 μL of dense mycelium glycerol stock of pSET152_ermE*p-based *Streptomyces* sp. SL203- or *Streptomyces* sp. SL294-generated strains, respectively, in a 100-mL Erlenmeyer flask and incubated at 28°C and 200 rpm for 5 days to produce a seed culture. Then, 250-mL baffled flasks containing 50 mL of MYM medium (4 g/L of maltose, 4 g/L of yeast extract, 10 g/L of malt extract, 1.9 g/L of morpholinepropanesulfonic acid [MOPS]) were inoculated with 3 mL of the well-grown cultures mentioned above. The fermentation was carried out at 200 rpm and 28°C for 7 days, after which the cultures were harvested and freeze-dried. Thirty milliliters of methanol was used to extract the freeze-dried material. Extraction was carried out at 200 rpm and room temperature for 1 h. The methanolic extracts were centrifuged at 4,000 rpm and room temperature for 10 min to get rid of debris before being concentrated under reduced pressure in 3 mL of methanol. The generated extracts were analyzed by LC-MS.

LC-MS analyses of the generated extracts were performed on a Vanquish Horizon ultrahigh-performance liquid chromatography (UHPLC) system coupled to the electrospray ionization (ESI) source of an LTQ Orbitrap Velos mass spectrometer (both from Thermo Fisher Scientific). Chromatographic and MS parameters were as described previously (78). The sum formulas of the detected ions were determined using Thermo Xcalibur 4.1.31.9 Qual browser based on the mass accuracy (Δ*m/z* ≤ 5 ppm) and isotopic pattern. Dereplication was accomplished with the aid of GNPS Library Search (79), the Dictionary of Natural Products version 31.1 (CRC Press, Taylor & Francis Group), and CAS SciFinder (American Chemical Society). MZmine 2 was used for comparing peak intensities between different samples or groups of samples (80).

For coprisidin A purification, five 250-mL flasks containing 50 mL of MYM medium were inoculated with *Streptomyces* sp. SL203 pSET152_ermE*p_SARP seeding culture and fermented as mentioned above. Cultures were centrifuged and the supernatant extracted with 3 volumes of ethyl acetate. The organic phase was evaporated under reduced-pressure conditions. The extract was dissolved in methanol and fractionated using a Luna C_18_, 10- by 250-mm, 5-μm HPLC semipreparative column (Phenomenex). Aqueous formic acid (0.1%) and acetonitrile were used as mobile phases A and B, respectively. The gradient used was 5 to 75% mobile phase B in 35 min followed by a washing (10 min at 95% mobile phase B) and reequilibration step (10 min at 10% mobile phase B). The flow rate was 4.7 mL/min, and a 254-nm wavelength was used for detection. Coprisidin A was eluted with a retention time of 25.4 min. Approximately 1 mg of coprisidin A was obtained.

For saccharoquinoline purification, 32 250-mL flasks containing 50 mL of MYM medium were inoculated with *Streptomyces* sp. SL294 pSET152_ermE*p_SARP seeding culture and fermented as mentioned above. Cultures were freeze-dried and extracted with methanol as previously described. The organic phase was evaporated under reduced-pressure conditions. The extract was dissolved in 200 mL of water and centrifuged to get rid of the debris; the clear supernatant was extracted with 3 volumes of chloroform. The organic phase was evaporated under reduced-pressure conditions. The extract was dissolved in methanol and fractionated using a Luna C_18_, 10- by 250-mm, 5-μm HPLC semipreparative column (Phenomenex). Aqueous formic acid (0.1%) and acetonitrile were used as mobile phases A and B, respectively. The gradient used was 45 to 95% mobile phase B in 25 min followed by a washing (10 min at 95% mobile phase B) and reequilibration step (10 min at 10% mobile phase B). The flow rate was 4.7 mL/min, and a 278-nm wavelength was used for detection. Saccharoquinoline was eluted with a retention time of 19.2 min. Approximately 1.5 mg of saccharoquinoline was obtained.

^1^H and ^13^C NMR spectra of coprisidin A in dimethyl sulfoxide (DMSO)-*d*_6_ at 298 K were recorded on an Avance III HDX 700 NMR spectrometer (700.40 MHz for ^1^H and 176.12 MHz for ^13^C) equipped with a 5-mm quadruple He cryoprobe QCI-F with *z* axis gradients and automatic tuning and matching accessory (Bruker BioSpin). ^1^H and ^13^C 1D as well as COSY, HSQC, and HMBC 2D NMR spectra of saccharoquinoline in DMSO-*d*_6_ at 298 K were recorded on an Avance NEO 600 NMR spectrometer (Bruker BioSpin) equipped with an N_2_ cryo probe Prodigy TCI with with z-gradient (600.18 MHz for ^1^H and 150.92 MHz for ^13^C). Chemical shifts were calibrated using the ^1^H residual solvent signal at δ of 2.50 and the ^13^C solvent signal at δ of 39.52.

### Data availability.

All the raw data and associated metadata for the different kinds of sequencing data reported and used in this study have been submitted to the BioProject database under accession number PRJNA896766. 16S rRNA sequences have been deposited to GenBank under accession numbers OP593123 to OP593300.
