# Supplementary material for: Secondary Metabolite Production Potential in a Microbiome of the Freshwater Sponge Spongilla lacustris
Source: Microbiol Spectr. 2023 Feb 2;11(2):e04353-22. doi: 10.1128/spectrum.04353-22 (PMC10100984; doi:10.1128/spectrum.04353-22)
Supplement: Supplemental file 1 — Tables S1 to S6 and Fig. S1 to S26. Download spectrum.04353-22-s0001.pdf, PDF file, 2.6 MB [file spectrum.04353-22-s0001.pdf]

Supplemental Material

Bacteria associated with the fresh-water sponge *Spongilla lacustris* and their potential to produce secondary metabolites revealed via genomics, metagenomics and genome mining

Sophie Graffius, Jaime Felipe Guerrero Garzón, Martin Zehl, Petra Pjevac, Rasmus Kirkegaard, Mathias Flieder, Alexander Loy, Thomas Rattei, Andrew Ostrovsky, Sergey B. Zotchev

Table S1. Summary of MAGs retrieved from *Spongilla lacustris* metagenome and their GTDB-based taxonomy.

MAG	marker lineage	completeness	contamination	GC, %	genome size	scaffolds	contigs	longest scaffold	longest contig	phylum_gtdb	class_gtdb	order_gtdb	family_gtdb	genus_gtdb	gtdb_closest	environment
Spongilla lacustris MAG01	k__Bacteria	78.42	0.00	35.4	475425	5	8	206783	113061	Patescibacteria	Paceibacteria	UBA9983	UBA2163	J119	GCA_003694545.1	iron-rich hot spring
Spongilla lacustris MAG02	k__Bacteria	100.00	8.62	59.0	3101799	39	41	295652	295652	Proteobacteria	Gammaproteobacteria	Burkholderiales	Burkholderiaceae	Limnohabitans	GCA_005789825.1	freshwater lake
Spongilla lacustris MAG03	p__Bacteroidetes	98.57	0.95	40.7	4628720	74	86	411324	376971	Bacteroidota	Bacteroidia	AKYH767	B-17BO	-	GCA_002342065.1	freshwater lake
Spongilla lacustris MAG04	c__Gammaproteobacteria	87.26	0.00	36.5	1716532	16	30	476275	476275	Proteobacteria	Gammaproteobacteria	GCA-002705445	GCA-002716945	-	NA	NA
Spongilla lacustris MAG05	p__Bacteroidetes	98.52	1.97	38.9	2493715	127	129	88460	88460	Bacteroidota	Bacteroidia	Chitinophagales	Chitinophagaceae	UBA1312	GCA_002482815.1	water, biofilm
Spongilla lacustris MAG06	k__Bacteria	69.80	4.31	28.6	587916	85	118	40569	18454	Patescibacteria	Paceibacteria	UBA9984	UBA5272	-	GCA_003518045.1	groundwater
Spongilla lacustris MAG07	k__Bacteria	89.89	0.55	32.0	2551601	156	209	87631	83425	Bacteroidota	Bacteroidia	Cytophagales	-	-	NA	NA
Spongilla lacustris MAG08	p__Bacteroidetes	86.70	0.99	34.1	2276483	203	219	51600	51600	Bacteroidota	Bacteroidia	Chitinophagales	Chitinophagaceae	Hydrotalea	NA	NA
Spongilla lacustris MAG09	c__Alphaproteobacteria	82.25	0.43	52.4	2299014	350	403	29586	27420	Proteobacteria	Alphaproteobacteria	Elsterales	-	-	NA	NA
Spongilla lacustris MAG10	c__Alphaproteobacteria	93.26	2.83	53.0	2719869	355	359	54419	54419	Proteobacteria	Alphaproteobacteria	-	-	-	NA	NA
Spongilla lacustris MAG11	p__Bacteroidetes	90.37	0.99	31.3	2716763	166	175	139035	139035	Bacteroidota	Bacteroidia	Chitinophagales	Chitinophagaceae	Sediminibacterium	NA	NA
Spongilla lacustris MAG12	k__Bacteria	78.98	0.07	67.7	2148011	426	429	26040	26040	Planctomycetota	Phycisphaerae	Phycisphaerales	SM1A02	SYIB01	GCA_005798965.1	freshwater lake
Spongilla lacustris MAG13	k__Bacteria	81.07	1.14	59.7	3350352	489	585	38525	38525	Planctomycetota	Phycisphaerae	Phycisphaerales	SM1A03	QWPT01	GCA_003671075.1	freshwater lake
Spongilla lacustris MAG14	o__Burkholderiales	54.73	0.06	44.0	1579445	270	278	26364	26364	Proteobacteria	Gammaproteobacteria	Burkholderiales	Burkholderiaceae	Symbiobacter	GCF_000477435.1	freshwater lake
Spongilla lacustris MAG15	k__Bacteria	87.23	1.35	53.1	1871277	100	163	165013	134855	Verrucomicrobiota	Verrucomicrobiae	Methylacidiphilales	UBA3015	UBA3015	GCA_001438005.1	Baltic Sea brackish surface water
Spongilla lacustris MAG16	k__Bacteria	96.77	1.08	48.9	1679970	91	119	152443	152443	Proteobacteria	Alphaproteobacteria	Elsterales	-	-	NA	NA
Spongilla lacustris MAG17	p__Bacteroidetes	96.85	1.23	36.1	3902886	134	235	123109	73227	Bacteroidota	Bacteroidia	Chitinophagales	Chitinophagaceae	-	NA	NA
Spongilla lacustris MAG18	p__Cyanobacteria	77.04	5.21	66.7	2492058	199	250	150199	94616	Cyanobacteria	Cyanobacteriia	PCC-6307	Cyanobiaceae	Cyanobium_A	GCA_007135755.1	hypersaline soda lake sediment
Spongilla lacustris MAG19	o__Burkholderiales	98.21	0.03	52.5	2643449	26	29	344907	344907	Proteobacteria	Gammaproteobacteria	Burkholderiales	Burkholderiaceae	Limnohabitans	GCA_005789825.1	freshwater lake
Spongilla lacustris MAG20	f__Flavobacteriaceae	98.23	3.49	31.2	4979690	313	339	202147	178244	Bacteroidota	Bacteroidia	Flavobacteriales	Flavobacteriaceae	Flavobacterium	NA	NA

Table S2. List of the oligonucleotide primers used in this work.

Name	Sequence (5´-3´)	Purpose
**ermEp_Fw**	GTCATCTAGAGGTGAACTTCGCACTTCG	Construction of pSET152_ermE*p plasmid
**ermEp_Rv**	TCCGGCTCGTATGTTGTG	Construction of pSET152_ermE*p plasmid
**ctg3_1243_Fw**	GTCAGCGGCCGCATGACCACGGCAATGCAG	Construction of pSET152_ermE*p_ctg3_1243 plasmid
**ctg3_1243_Rv**	GTCAGAATTCGCGACGACCTGGCCTGAC	Construction of pSET152_ermE*p_ctg3_1243 plasmid

Table S3. BGCs identified in the genome of *Streptomyces* sp. SL203 with antiSMASH version 6.1.1.

BGC	Type	From	To	Most similar known BGC	Similarity	Presence in another bacterium	Found and assigned NP
3.1	butyrolactone	237,860	246,265	-	-	*Streptomyces* sp. PAMC 26508	-
3.2	NRPS	319,354	372,367	rimosamide	21%	*Streptomyces* sp. PAMC 26508	detoxin S_1
3.3	PKSII, terpene	514,723	586,172	spore pigment	83%	*Streptomyces* sp. PAMC 26508	-
3.4	melanin	612,086	622,550	melanin	100%	*Streptomyces pratensis* ATCC 33331	-
3.5	PKSIII	719,172	760,230	-	-	*Streptomyces pratensis* ATCC 33331	-
3.6	RiPP-like	919,628	926,490	-	-	*Streptomyces pratensis* ATCC 33331	-
3.7	terpene, ectoine	957,073	977,440	ectoine	100%	*Streptomyces pratensis* ATCC 33331	ectoine
3.8	terpene	1,258,237	1,278,832	-	-	*Streptomyces pratensis* ATCC 33331	-
3.9	PKSII, PKS-like	1,323,735	1,396,271	cinerubin B	25%	*Streptomyces pratensis* ATCC 33331	coprisidin A/B
3.10	ectoine	1,758,007	1,768,405	ectoine	100%	*Streptomyces pratensis* ATCC 33331	ectoine
3.11	terpene	2,230,079	2,249,836	-	-	*Streptomyces* sp. PAMC 26508	-
3.12	lanthipeptide class III	2,690,330	2,712,981	-	-	*Streptomyces* sp. CB01580	-
3.13	siderophore	2,757,623	2,769,263	desferrioxamine B/E	83%	*Streptomyces* sp. PAMC 26508	(dehydroxy)nocardamine
3.14	NRPS, PKSI, NRPS-like	3,413,177	3,467,764	-	-	*Streptomyces pratensis* ATCC 33331	-
3.15	butyrolactone	3,667,911	3,678,837	-	-	*Streptomyces pratensis* ATCC 33331	-
3.16	RiPP-like	5,333,512	5,342,409	-	-	*Streptomyces* sp. PAMC 26508	-
3.17	terpene	5,500,170	5,520,316	-	-	*Streptomyces pratensis* ATCC 33331	-
3.18	siderophore	5,585,671	5,600,255	-	-	*Streptomyces pratensis* ATCC 33331	-
3.19	NRPS	5,872,256	5,927,100	-	-	*Streptomyces pratensis* ATCC 33331	-
3.20	lanthipeptide class I	5,947,894	5,974,342	-	-	*Streptomyces* sp. ADI97-07	-
3.21	RiPP-like	6,094,486	6,103,893	-	-	*Streptomyces* sp. ADI97-07	-
3.22	PKSI	6,320,692	6,408,208	sceliphrolactam	88%	*Streptomyces pratensis* ATCC 33331	sceliphrolactam
3.23	terpene	6,747,935	6,773,426	hopene	69%	*Streptomyces anulatus* ATCC 11523	-
3.24	β-lactam	6,831,354	6,850,351	clavulanic acid	20%	*Streptomyces* sp. PAMC 26508	-
3.25	RiPP-like	6,920,994	6,931,518	-	-	*Streptomyces pratensis* ATCC 33331	-
3.26	terpene	7,070,847	7,096,339	isorenieratene	100%	*Streptomyces pratensis* ATCC 33331	-
3.27	NRPS	7,110,740	7,160,490	coelichelin	90%	*Streptomyces pratensis* ATCC 33331	coelichelin
3.28	β-lactam, PKSI, NRPS	7,277,936	7,418,487	carbapenem MM4550	55%	*Streptomyces pratensis* ATCC 33331	-

Table S4. BGCs identified in the genome of *Streptomyces* sp. SL294 with antiSMASH version 6.1.1.

BGC	Type	From	To	Most similar known BGC	Similarity	Presence in another bacterium	Found and assigned NP
3.1	NRPS, PKSI, β-lactam	128,640	268,174	carbapenem MM4550	58%	*Streptomyces pratensis* ATCC 33331	-
3.2	NRPS	385,716	436,620	coelichelin	90%	*Streptomyces pratensis* ATCC 33331	coelichelin
3.3	terpene	451,251	475,851	isorenieratene	100%	*Streptomyces pratensis* ATCC 33331	-
3.4	RiPP-like	616,557	625,518	-	-	*Streptomyces pratensis* ATCC 33331	-
3.5	β-lactam	692,238	715,732	clavulanic acid	20%	*Streptomyces microflavus* CG 893	-
3.6	terpene	772,612	799,206	hopene	69%	*Streptomyces pratensis* ATCC 33331	-
3.7	PKSI	1,138,589	1,233,400	sceliphrolactam	88%	*Streptomyces pratensis* ATCC 33331	sceliphrolactam
3.8	RiPP-like	1,441,862	1,453,154	-	-	*Streptomyces* sp. ADI97-07	-
4.1	lanthipeptide class I	73,205	99,734	-	-	*Streptomyces* sp. ADI97-07	-
4.2	NRPS	120,528	175,401	-	-	*Streptomyces* sp. S501	-
4.3	siderophore	448,476	461,607	-	-	*Streptomyces pratensis* ATCC 33331	-
4.4	terpene	527,356	547,053	-	-	*Streptomyces pratensis* ATCC 33331	-
4.5	RiPP-like	705,268	714,816	-	-	*Streptomyces pratensis* ATCC 33331	-
4.6	butyrolactone	2,368,883	2,379,809	-	-	*Streptomyces pratensis* ATCC 33331	-
4.7	NRPS-like, PKSI, NRPS	2,579,964	2,634,827	-	-	*Streptomyces pratensis* ATCC 33331	-
4.8	siderophore	3,279,372	3,291,153	desferrioxamine B/E	83%	*Streptomyces microflavus* CG 893	(dehydroxy)nocardamine
4.9	lanthipeptide class III, lanthipeptide class II	3,335,681	3,366,663	-	-	*Streptomyces microflavus* CG 893	-
4.10	terpene	3,798,845	3,818,603	-	-	*Streptomyces microflavus* CG 893	-
4.11	ectoine	4,281,704	4,290,312	ectoine	100%	*Streptomyces pratensis* ATCC 33331	ectoine
4.12	PKSII, PKS-like	4,652,529	4,725,065	cinerubin B	25%	*Streptomyces pratensis* ATCC 33331	coprisidin A/B
4.13	terpene	4,769,968	4,790,570	-	-	*Streptomyces pratensis* ATCC 33331	-
4.14	terpene, ectoine	5,070,871	5,092,300	ectoine	100%	*Streptomyces pratensis* ATCC 33331	ectoine
4.15	RiPP-like	5,120,490	5,130,717	-	-	*Streptomyces pratensis* ATCC 33331	-

Table S5. ^{1}H (700 MHz, DMSO-d_6) and ^{13}C NMR data (176 MHz, DMSO-d_6) of **8** in comparison with literature data for coprisidin A (δ in ppm).

	**8**		Coprisidin A[a]	
Position	δ_H (J in Hz)	δ_C, type	δ_H (J in Hz)	δ_C, type
1	—	n.d.	—	183.7, C
1a	—	123.77, C	—	123.6, C
2	—	n.d.	—	147.2, C
3	—	n.d.	—	155.5, C
3-OH	10.61, br s	—	10.86, s	—
4	—	n.d.	—	131.0, C
5	7.44, s	120.54, CH	7.47, s	120.4, CH
5a	—	n.d.	—	122.3, C
6	—	n.d.	—	179.7, C
7	—	n.d.	—	154.1, C
7-OH	10.61, br s	—	10.60, br s	—
8	—	n.d.	—	124.6, C
9	2.37, t (7.5)	24.78, CH_2	2.38, t (7.0)	24.8, CH_2
10	1.40, qt (7.5, 7.4)	21.18, CH_2	1.41, qt (7.0, 7.0)	21.2, CH_2
11	0.87, t (7.4)	14.09, CH_3	0.88, t (7.0)	14.1, CH_3
12	3.76, s	n.d.	3.77, s	61.5, CH_3
NH	8.77, s	—	8.81, s	—
2′	—	170.35, C	—	170.3, C
3′	5.99, s	53.88, CH	6.00, s	53.8, CH
3′a	—	116.34, C	—	116.4, C
4′	—	155.24, C	—	155.3, C
4′-OH	9.64, s	—	9.80, s	—
5′	6.86, d (7.5)	113.93, CH	6.85, d (7.5)	113.9, CH
6′	7.31, dd (8.1, 7.5)	133.73, CH	7.32, dd (7.5, 7.5)	133.9, CH
7′	6.79, d (8.1)	115.02, CH	6.81, d (7.5)	115.2, CH
7′a	—	n.d.	—	149.3, C

[a] Um, S., Bach, D. H., Shin, B., Ahn, C. H., Kim, S. H., Bang, H. S., Oh, K. B., Lee, S. K., Shin, J. and Oh, D. C., 2016. Naphthoquinone-Oxindole Alkaloids, Coprisidins A and B, from a Gut-Associated Bacterium in the Dung Beetle, Copris tripartitus. Organic letters, 18(22), 5792–5795. *https://doi.org/10.1021/acs.orglett.6b02555*

Table S6. ^{1}H (600 MHz, DMSO-d_6) and ^{13}C NMR data (151 MHz, DMSO-d_6) of **14** in comparison with literature data for saccharoquinoline (δ in ppm).

	**14**		Saccharoquinoline[a]	
Position	δ_H (J in Hz)	δ_C, type	δ_H (J in Hz)	δ_C, type
1	1.84, brd (12.5); 1.00, td (13.0, 2.9)	38.49, CH_2	1.83, d (14.3); 0.99, brd;	38.5, CH_2
2	1,65[b,c], m; 1,45[b,c], m	18.07, CH_2	1.66, m; 1.44, m	18.0, CH_2
3	1.39[b], brd; 1.17[b,c], m	41.36, CH_2	1.39; 1.18	41.3, CH_2
4	—	32.88, C	—	32.8, C
5	1.09, d (11.8)	55.33, CH	1.08	55.3, CH
6	1,74[b,c], m; 1,43[b,c], m	19.27, CH_2	1.74, m; 1.42, m	19.3, CH_2
7	2.14, m; 1,73[b,c], m	40.33, CH_2	2.15, dt (12.3, 3.6); 1.73	40.3, CH_2
8	—	78.17, C	—	78.1, C
9	1.67[b], dd (12.8, 5.1)	51.26, CH	1.67	51.2, CH
10	—	36.60, C	—	36.6, C
11	2.91, dd (16.4, 5.0); 2.70, dd (16.2, 13.1)	17.90, CH_2	2.91, dd (16.8, 13.1); 2.69, dd (16.8, 5.2)	17.9, CH_2
12	0.85, s	21.47, CH_3	0.85, s	21.5, CH_3
13	0.91, s	33.22, CH_3	0.90, s	33.2, CH_3
14	0.94, s	14.58, CH_3	0.94, s	14.6, CH_3
15	1.18[b], s	20.37, CH_3	1.18, s	20.3, CH_3
1′	—	104.86, C	—	104.9, C
2′	—	122.84, C	—	122.8, C
3′	—	133.60, C	—	133.6, C
4′	—	135.04, C	—	135.0, C
5′	—	136.96, C	—	136.9, C
6′	—	145.80, C	—	145.8, C
7′	8.40, d (8.6)	132.09, CH	8.40, d (8.6)	132.1, CH
8′	7.92, d (8.6)	116.93, CH	7.92, d (8.6)	116.9, CH
9′	—	140.90, C	—	140.8, C
10′	—	165.58, C	—	165.6, C

[a] Le, T. C., Lee, E. J., Lee, J., Hong, A., Yim, C. Y., Yang, I., Choi, H., Chin, J., Cho, S. J., Ko, J., Hwang, H., Nam, S. J. and Fenical, W., 2019. Saccharoquinoline, a Cytotoxic Alkaloidal Meroterpenoid from Marine-Derived Bacterium *Saccharomonospora* sp. *Marine Drugs*, *17*(2), 98. https://doi.org/10.3390/md17020098
[b] Overlapping signal. [c] Value determined from the HSQC spectrum.

Figure S1.

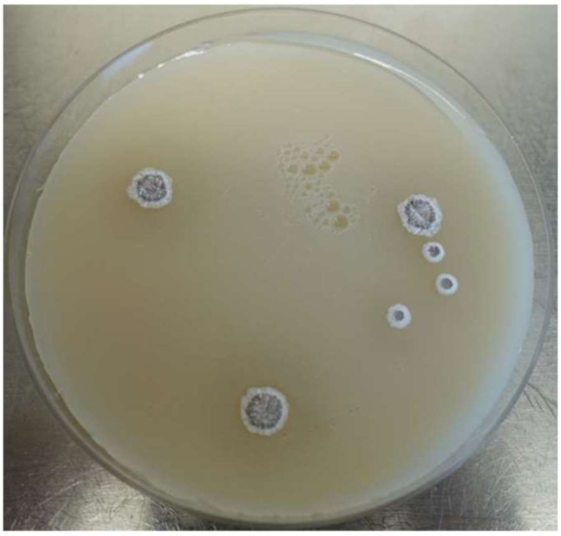

Streptomyces sp. SL203

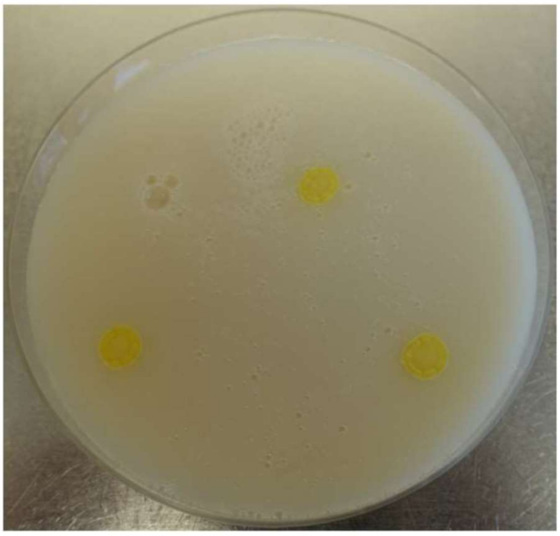

Streptomyces sp. SL294

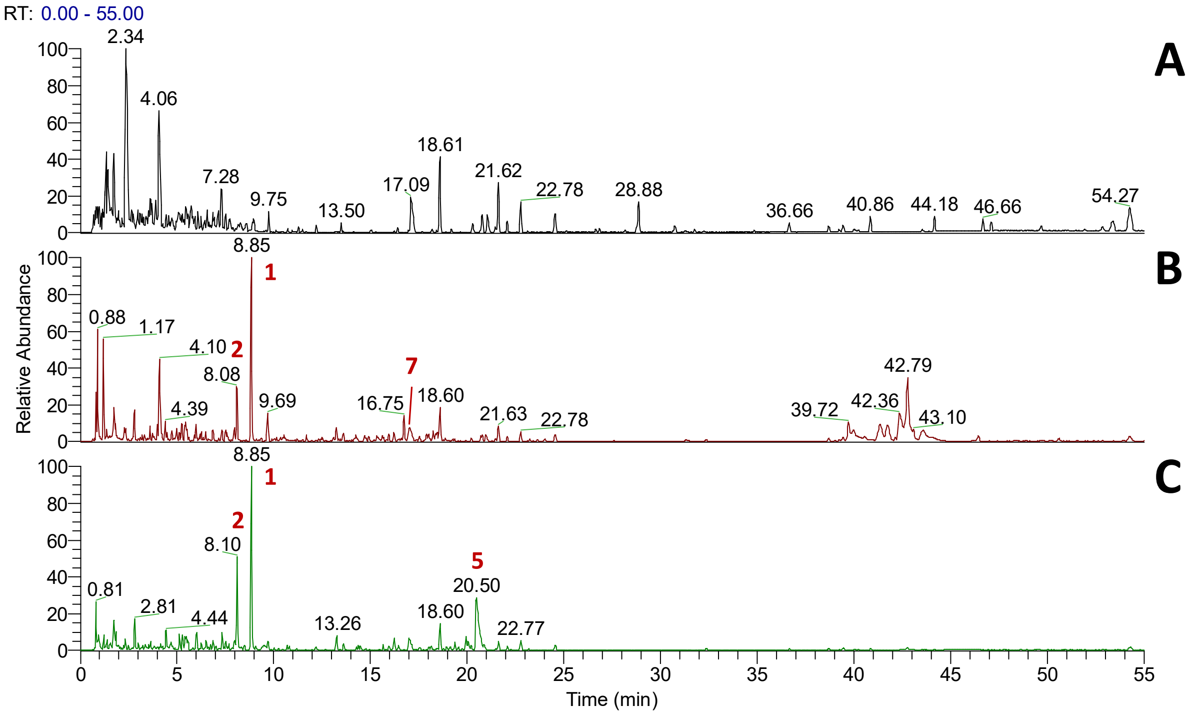

Figure S2. Base peak chromatograms (*m/z* 140-2000) obtained by LC-MS in positive ion mode of the extracts from SM17 medium control (A), strain SL203 pSET152_ermE*p grown in SM17 medium (B), and strain SL294 pSET152_ermE*p grown in SM17 medium (C). The peaks corresponding to nocardamine (**1**), dehydroxynocardamine (**2**), coproporphyrin (**5**), and detoxin S_1 (**7**) are labelled.

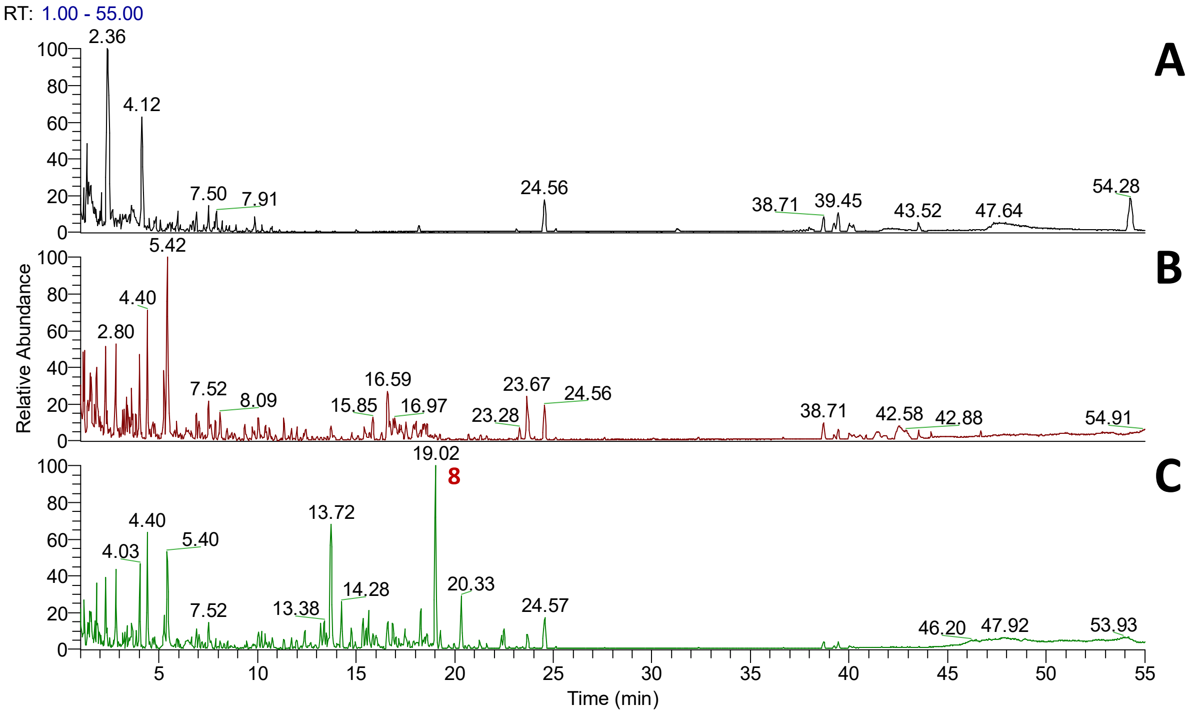

Figure S3. Base peak chromatograms (*m/z* 140-2000) obtained by LC-MS in positive ion mode of the extracts from MYM medium control (A), strain SL203 pSET152_ermE*p grown in MYM medium (B), and strain SL203 pSET152_ermE*p_ctg3_1243 grown in MYM medium (C). The peak corresponding to coprisidin A (**8**) is labelled.

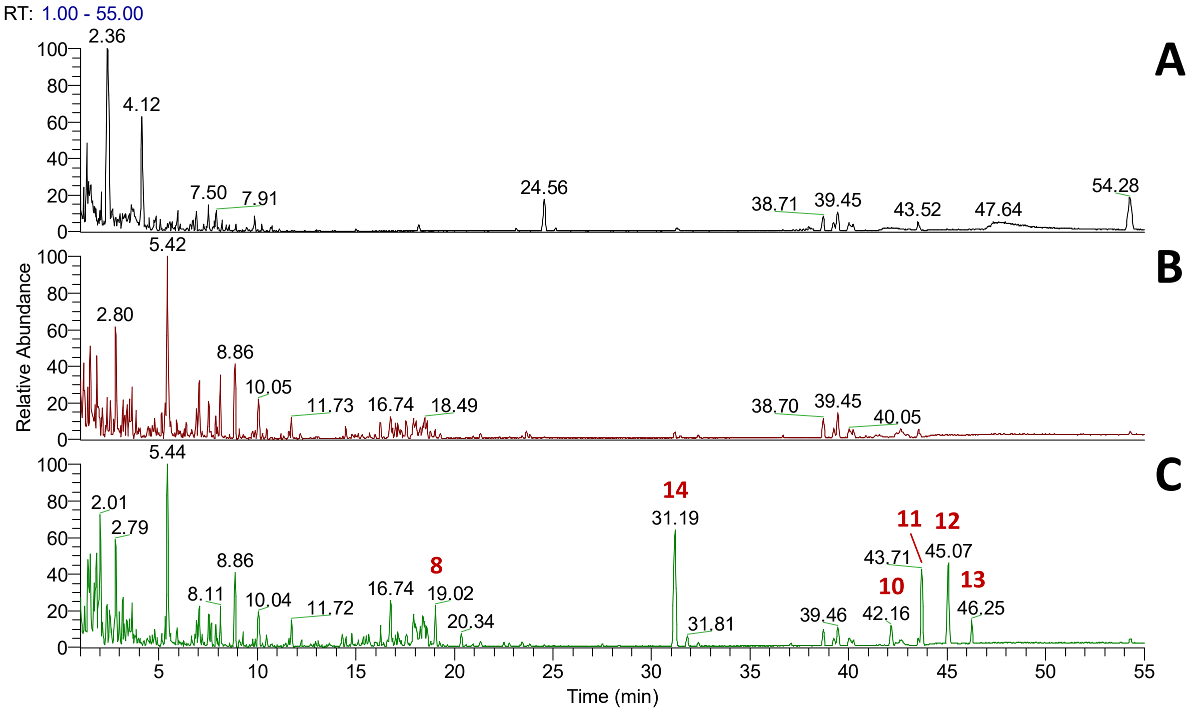

Figure S4. Base peak chromatograms (*m/z* 140-2000) obtained by LC-MS in positive ion mode of the extracts from MYM medium control (A), strain SL294 pSET152_ermE*p grown in MYM medium (B), and strain SL294 pSET152_ermE*p_ctg3_1243 grown in MYM medium (C). The peaks corresponding to coprisidin A (**8**), saccharoquinoline (**14**) and the congeners nonactin (**10**), monactin (**11**), isodinactin (**12**), and either trinactin or isotrinactin (**13**) are labelled.

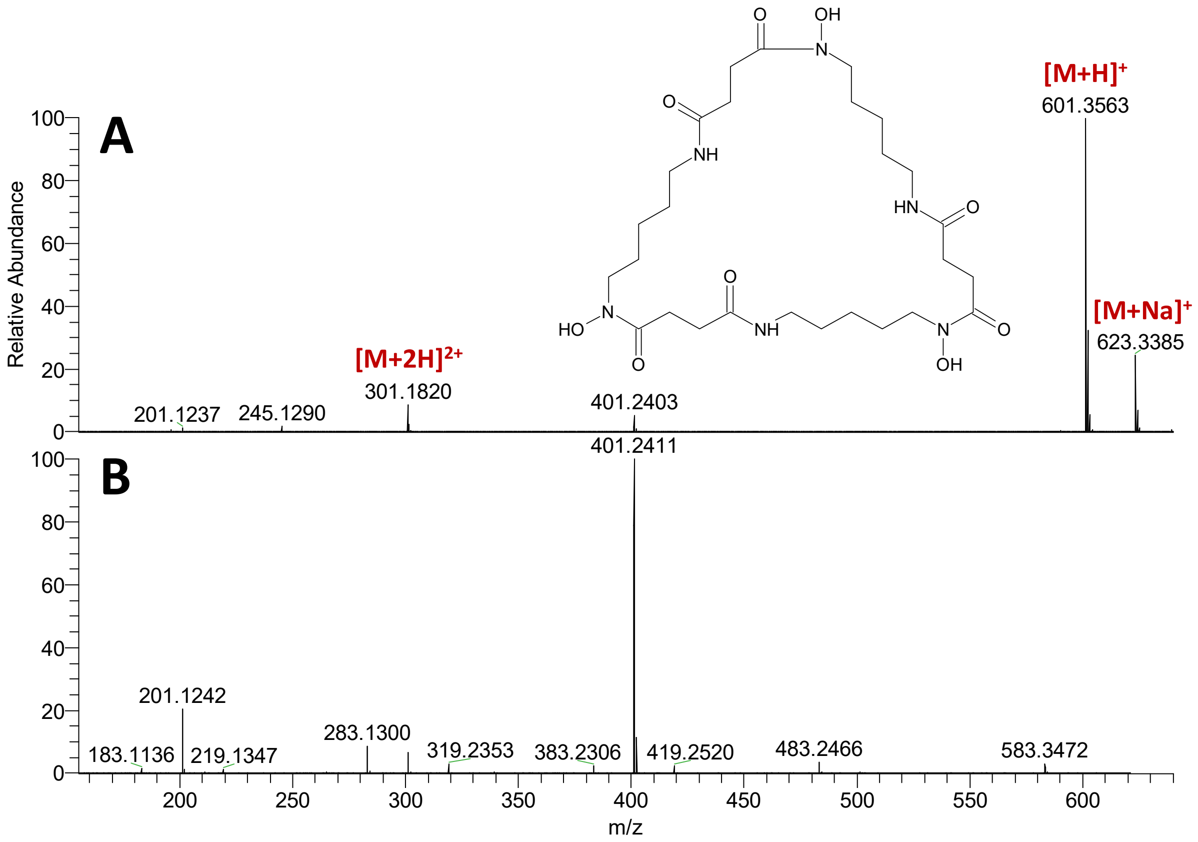

Figure S5. High resolution ESI mass spectrum of nocardamine (**1**) at R_t 8.76-8.94 min (A) and high resolution MS/MS spectrum of its $[M+H]^+$ ion at *m/z* 601.3563 (B).

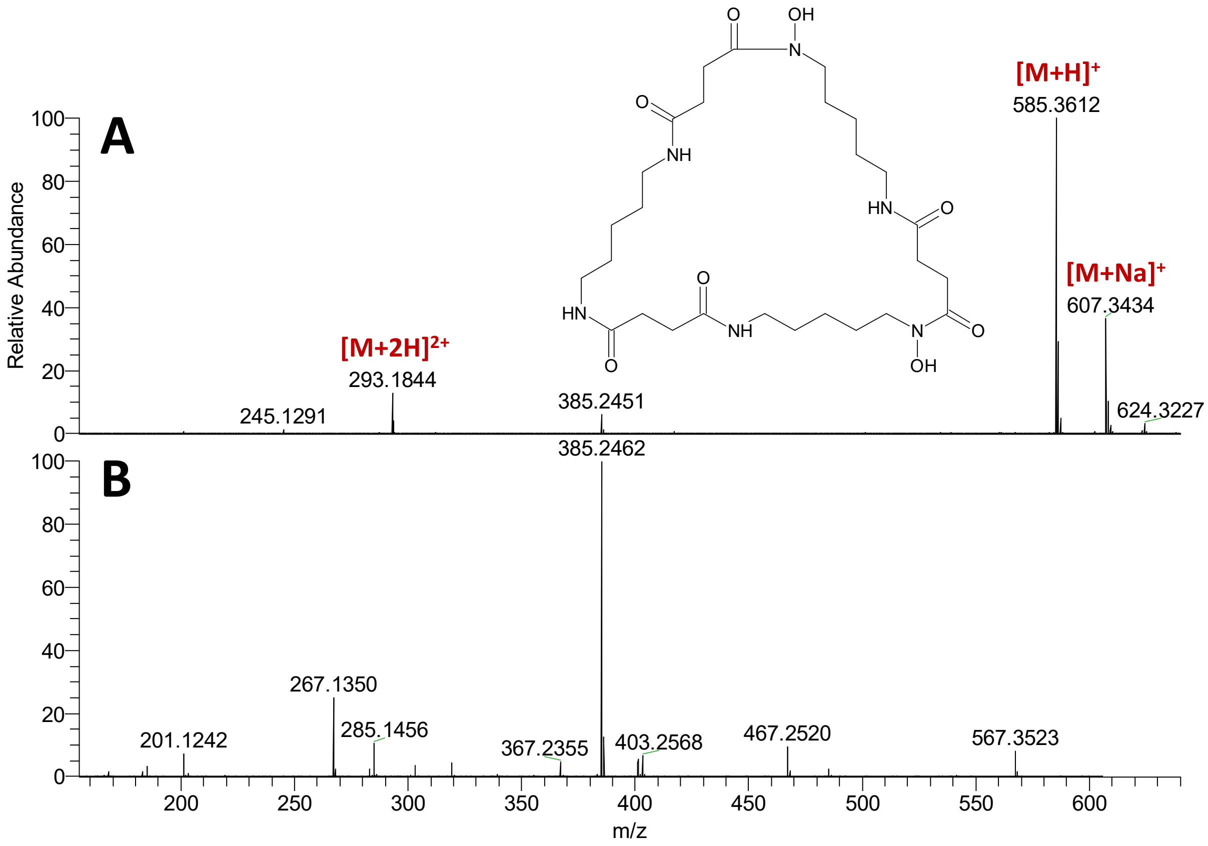

Figure S6. High resolution ESI mass spectrum of dehydroxynocardamine (**2**) at R_t 8.01-8.21 min (A) and high resolution MS/MS spectrum of its $[M+H]^+$ ion at *m/z* 585.3612 (B).

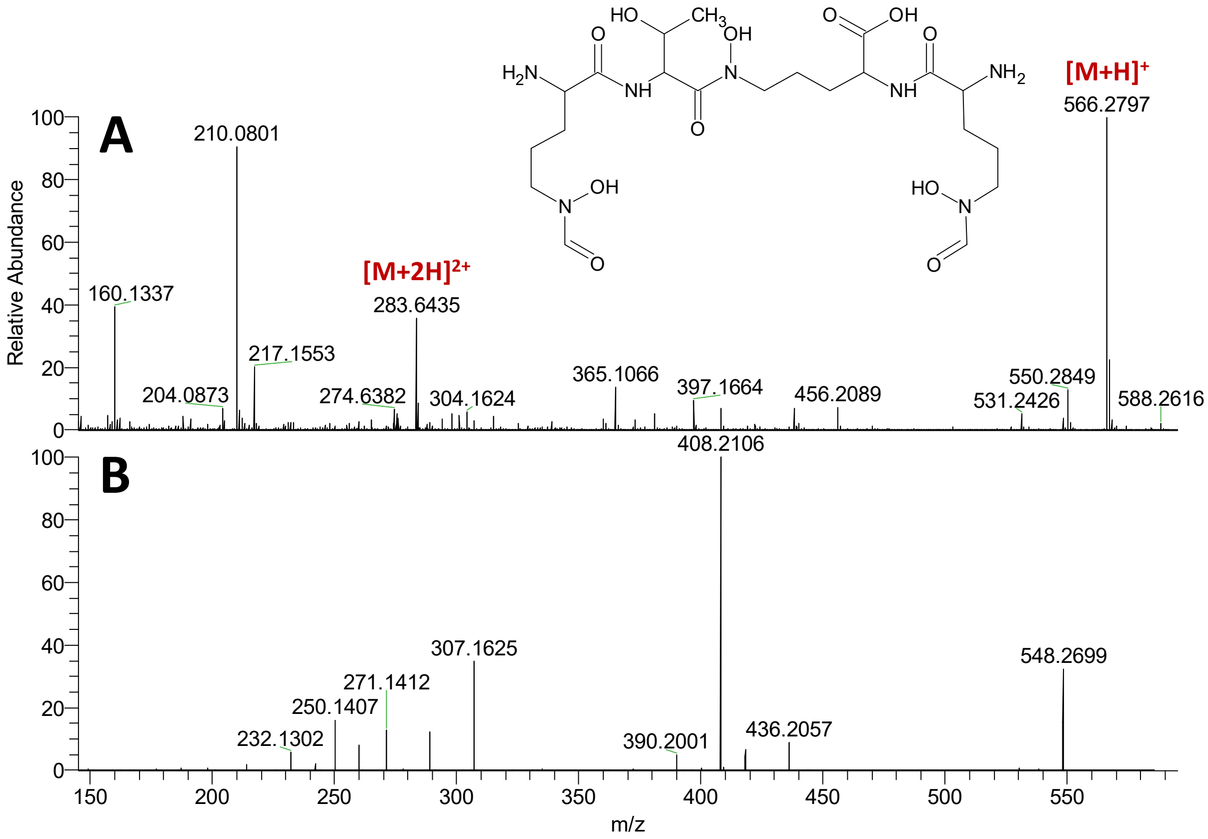

Figure S7. High resolution ESI mass spectrum of coelichelin (**3**) at R_t 1.10-1.13 min (A) and high resolution MS/MS spectrum of its $[M+H]^+$ ion at *m/z* 566.2797 (B).

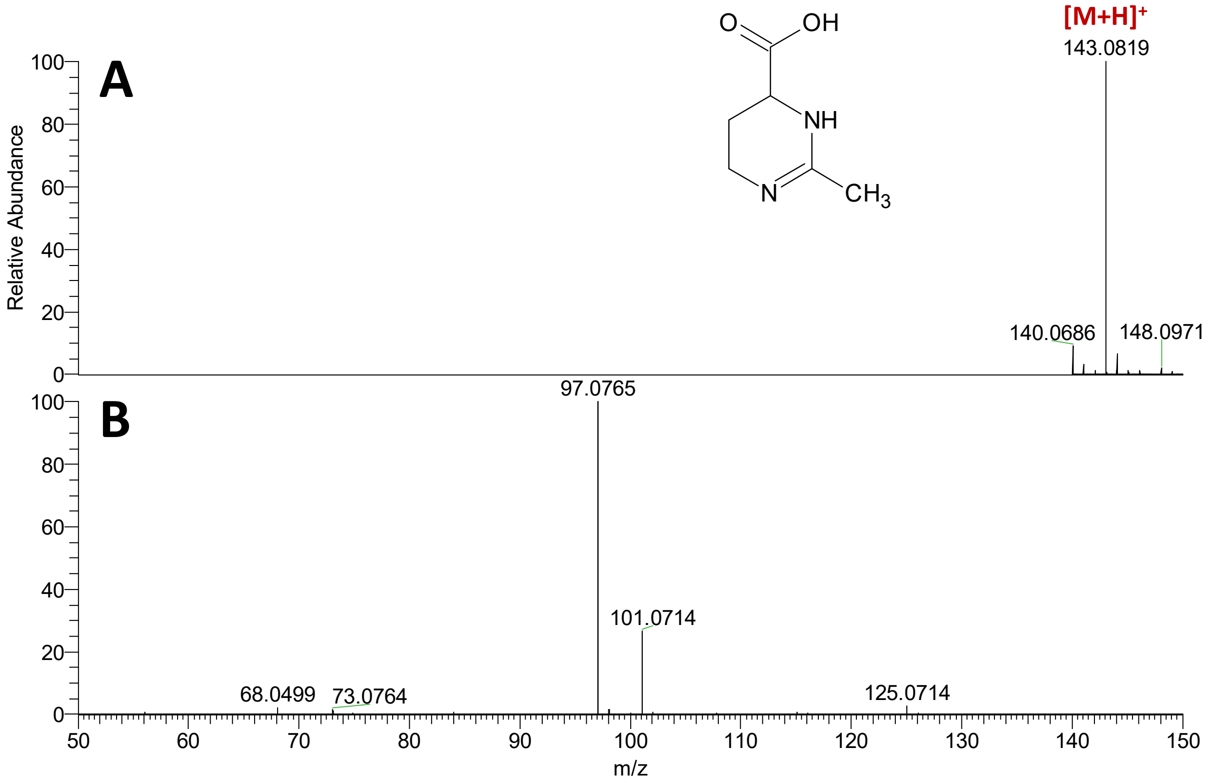

Figure S8. High resolution ESI mass spectrum of ectoine (**4**) at R_t 0.80-0.91 min (A) and high resolution MS/MS spectrum of its $[M+H]^+$ ion at *m/z* 143.0819 (B).

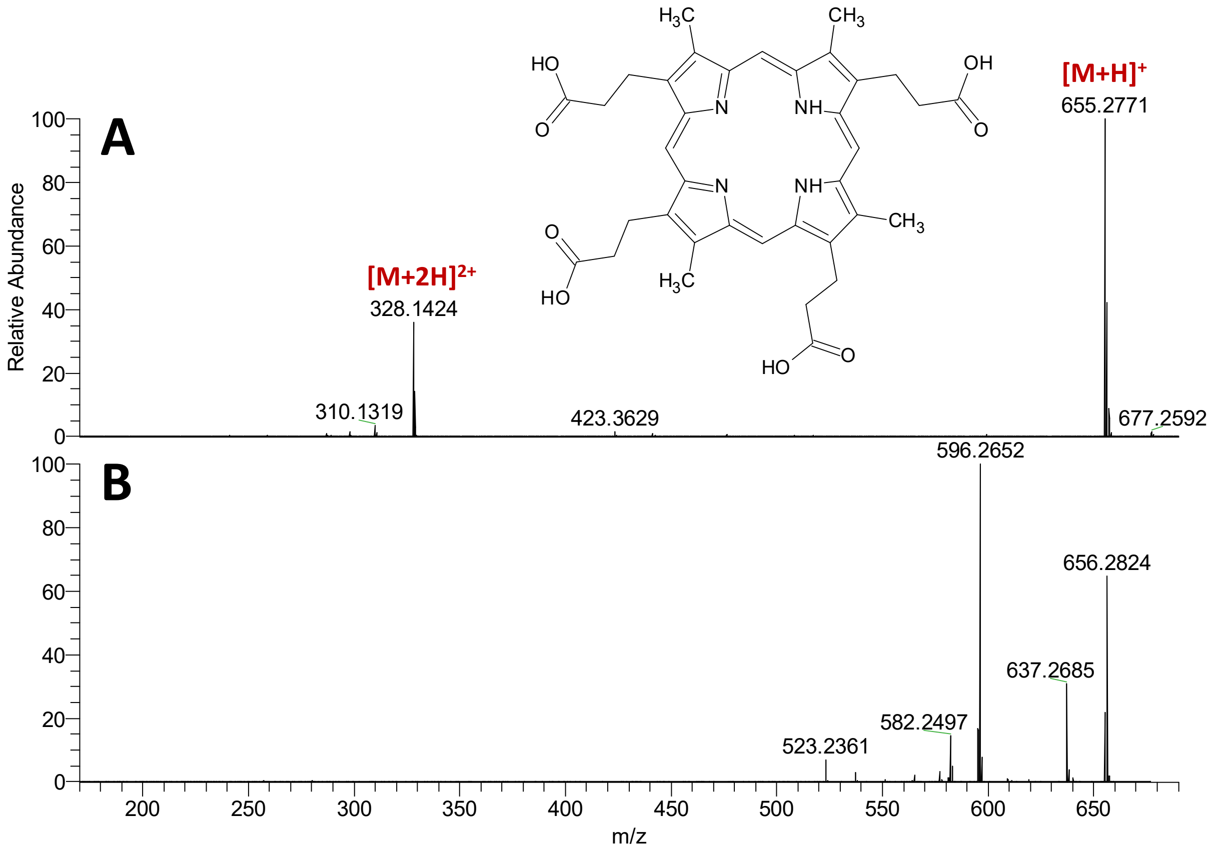

Figure S9. High resolution ESI mass spectrum of coproporphyrin (**5**) at R_t 20.41-20.82 min (A) and high resolution MS/MS spectrum of its $[M+H]^+$ ion at *m/z* 655.2771 (B).

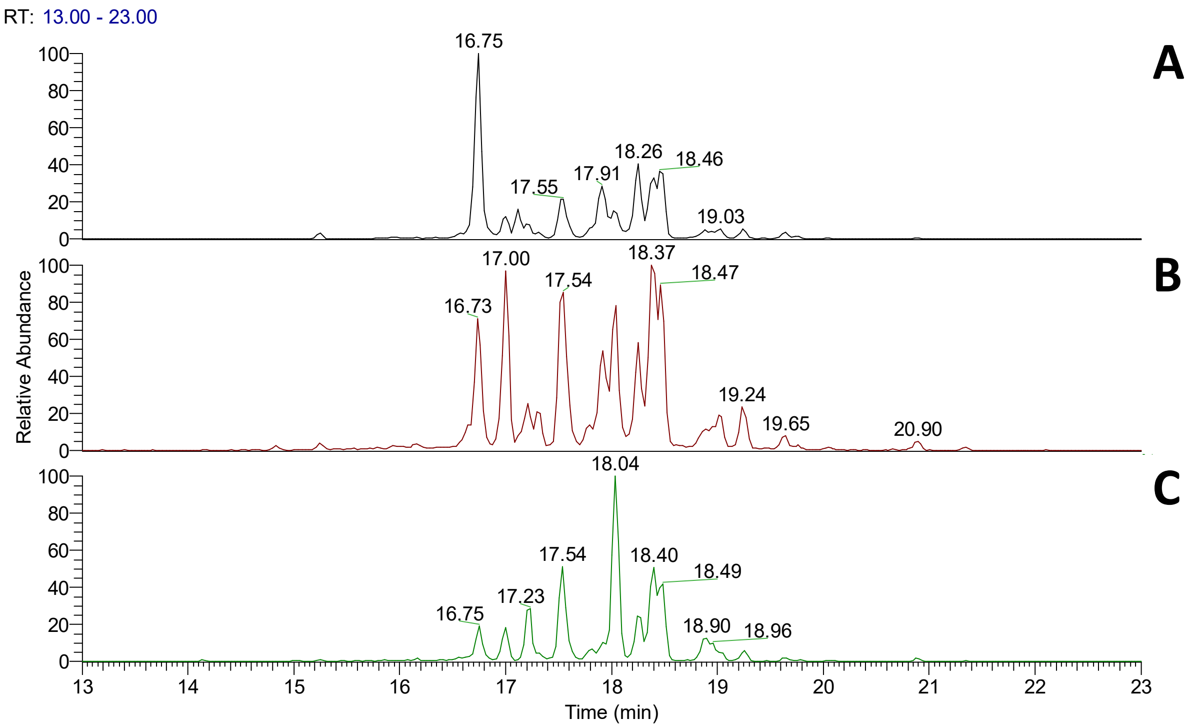

Figure S10. Extracted ion chromatograms (*m/z* 482.2537±0.0024) obtained by LC-MS in positive ion mode of the extracts from strain SL203 pSET152_ermE*p grown in SM17 medium (A), strain SL203 pSET152_ermE*p grown in MYM medium (B), and strain SL294 pSET152_ermE*p grown in SM17 medium (C). This *m/z* value corresponds to the $[M+H]^+$ ion of the various sceliphrolactam isomers.

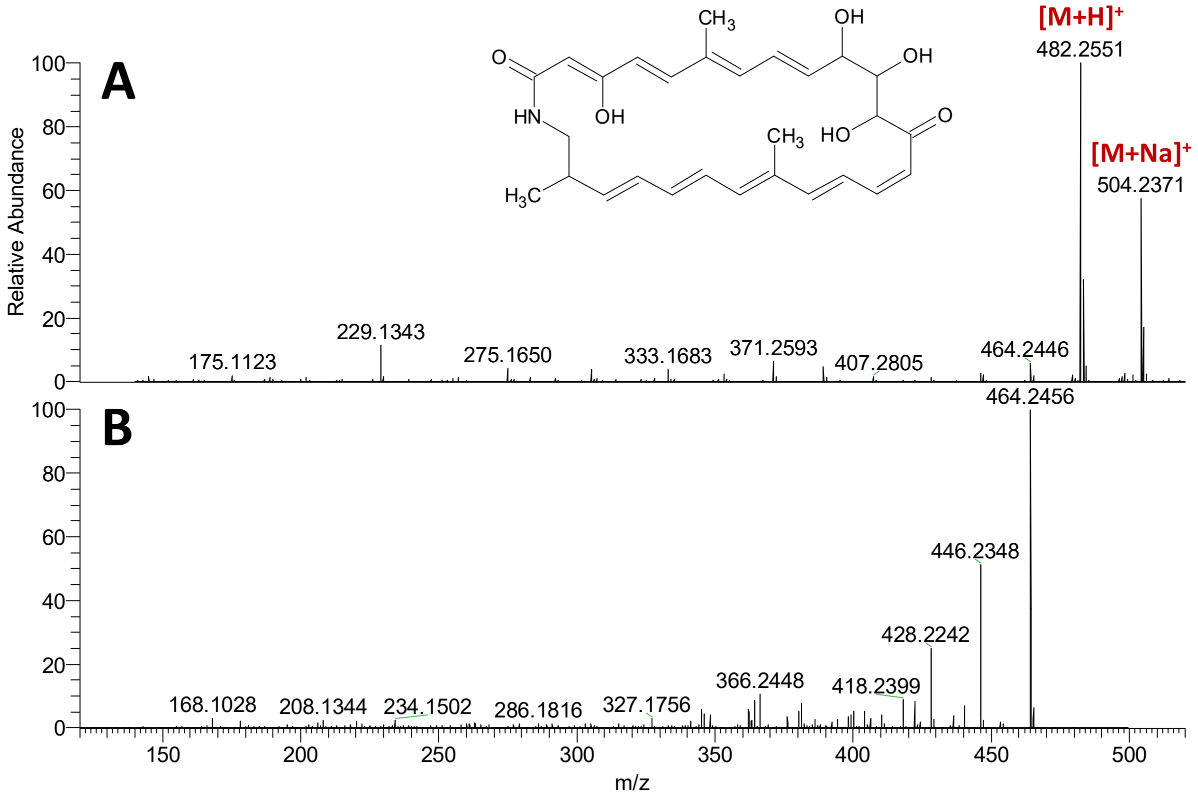

Figure S11. High resolution ESI mass spectrum of the sceliphrolactam isomer eluting at R_t 16.63-16.83 min (A) and high resolution MS/MS spectrum of its $[M+H]^+$ ion at *m/z* 482.2551 (B). The structure shown is for sceliphrolactam as representative for the different isomers.

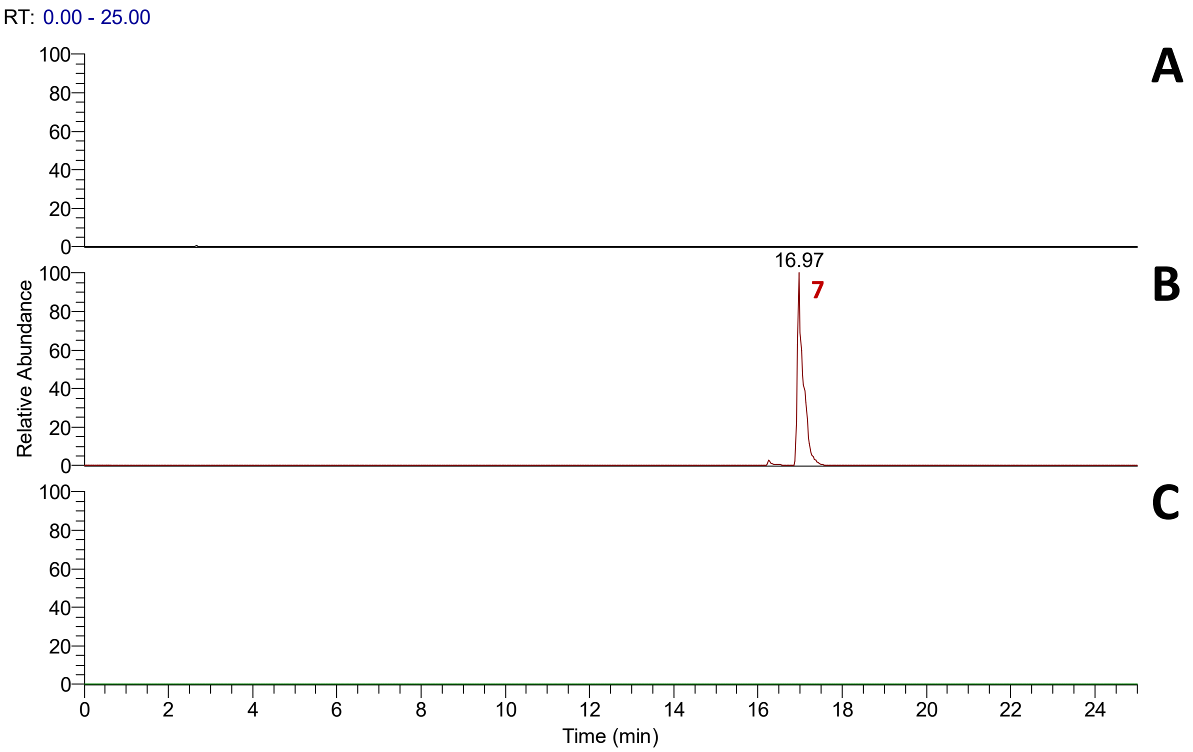

Figure S12. Extracted ion chromatograms (*m/z* 518.3225±0.0026) obtained by LC-MS in positive ion mode of the extracts from SM17 medium control (A), strain SL203 pSET152_ermE*p grown in SM17 medium (B), and strain SL294 pSET152_ermE*p grown in SM17 medium (C). This *m/z* value corresponds to the $[M+H]^+$ ion of detoxin S_1 (**7**).

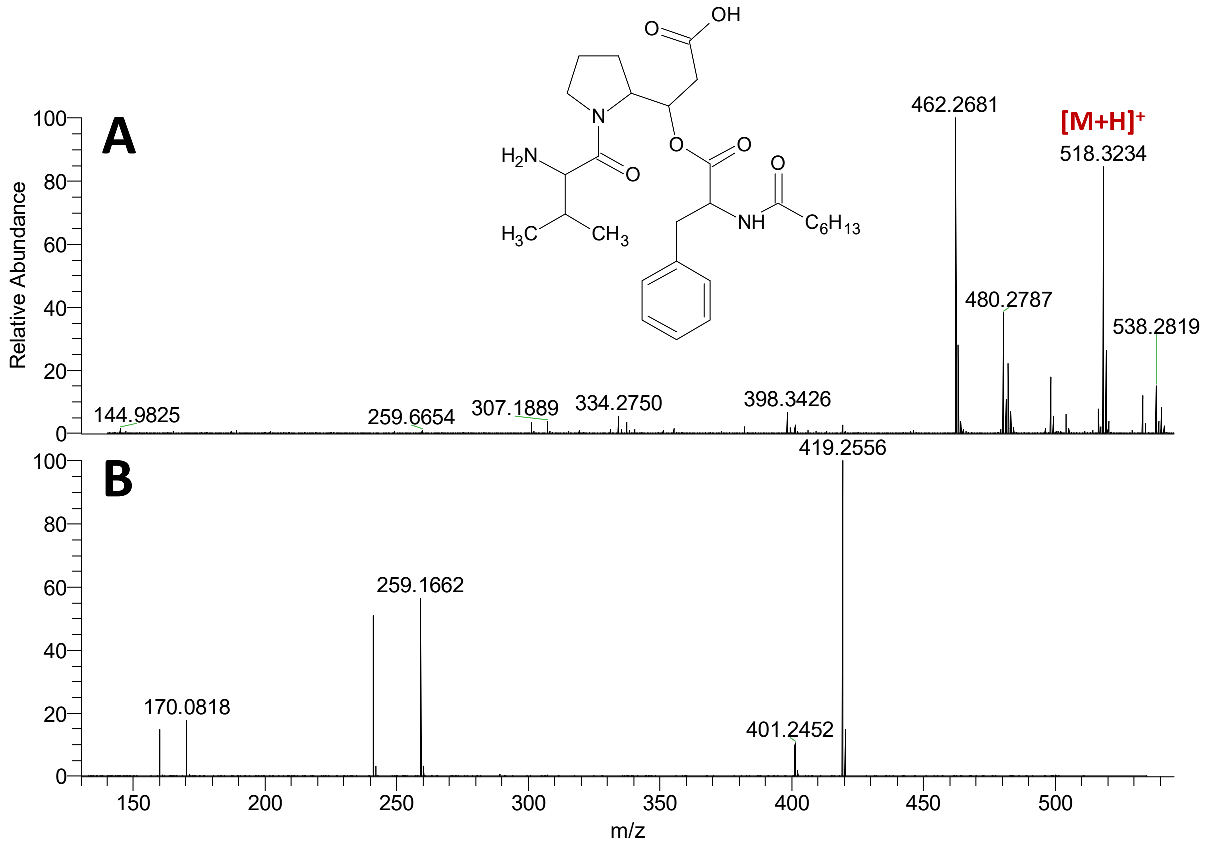

Figure S13. High resolution ESI mass spectrum of detoxin S_1 (**7**) at R_t 16.86-17.31 min (A) and high resolution MS/MS spectrum of its $[M+H]^+$ ion at *m/z* 518.3234 (B). The structure of the *N*-acyl chain attached to the Phe is not known.

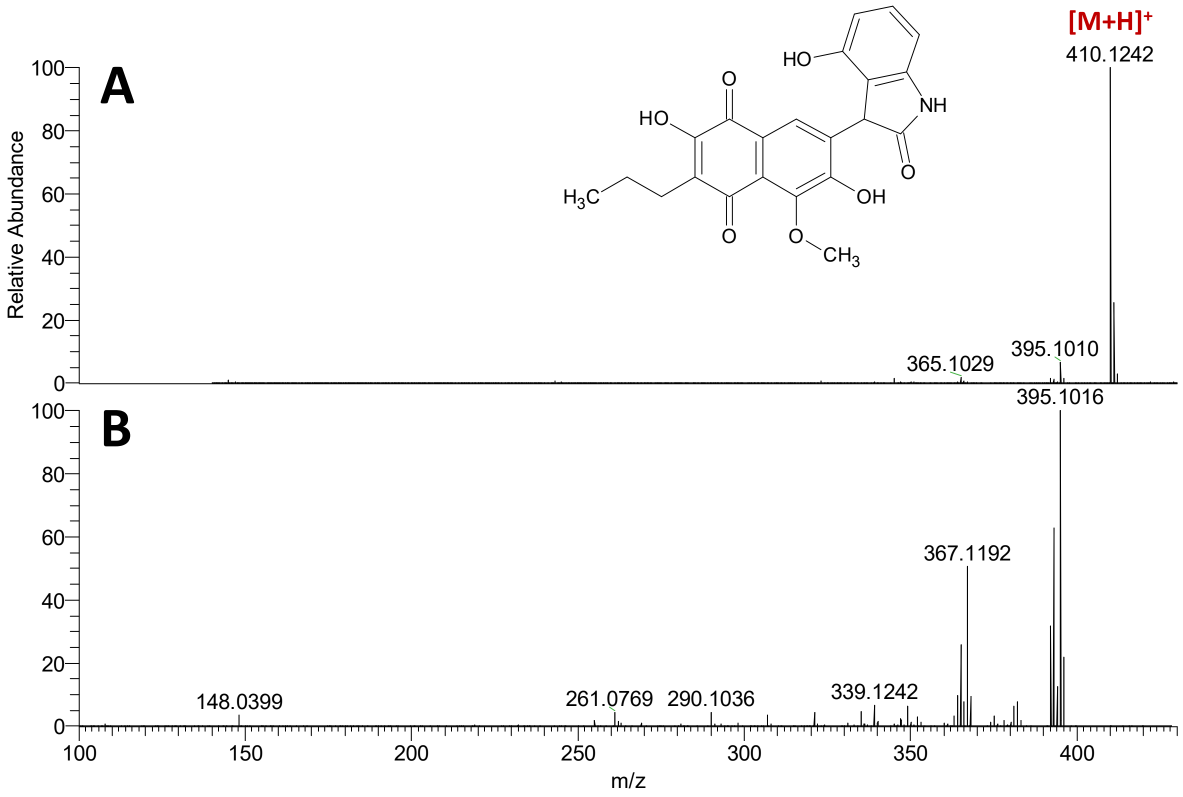

Figure S14. High resolution ESI mass spectrum of coprisidin A (**8**) at R_t 18.94-19.11 min (A) and high resolution MS/MS spectrum of its $[M+H]^+$ ion at *m/z* 410.1242 (B).

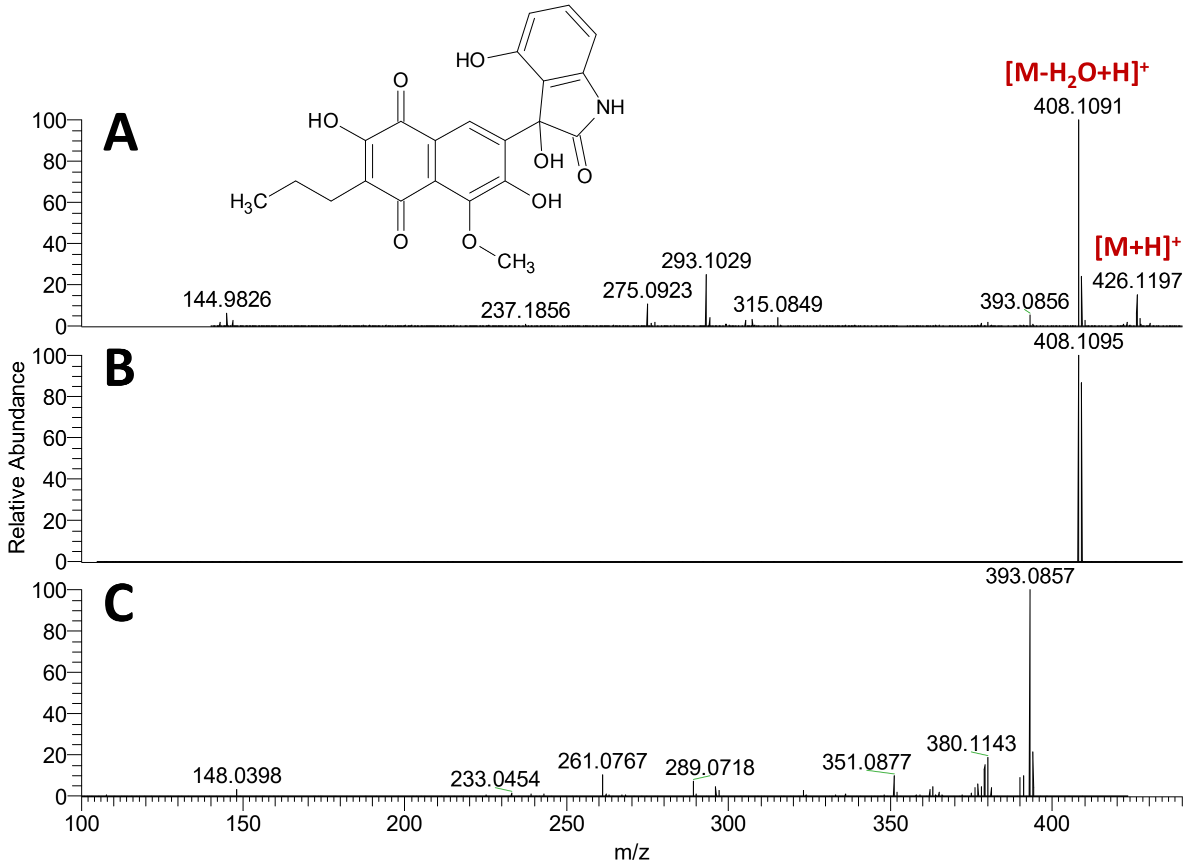

Figure S15. High resolution ESI mass spectrum of coprisidin B (**9**) at R_t 17.41-17.53 min (A), high resolution MS/MS spectrum of its $[M+H]^+$ ion at *m/z* 426.1197 (B), and high resolution MS/MS spectrum of its $[M\text{-}H_2O+H]^+$ ion (formed via in source decay) at *m/z* 408.1091 (B).

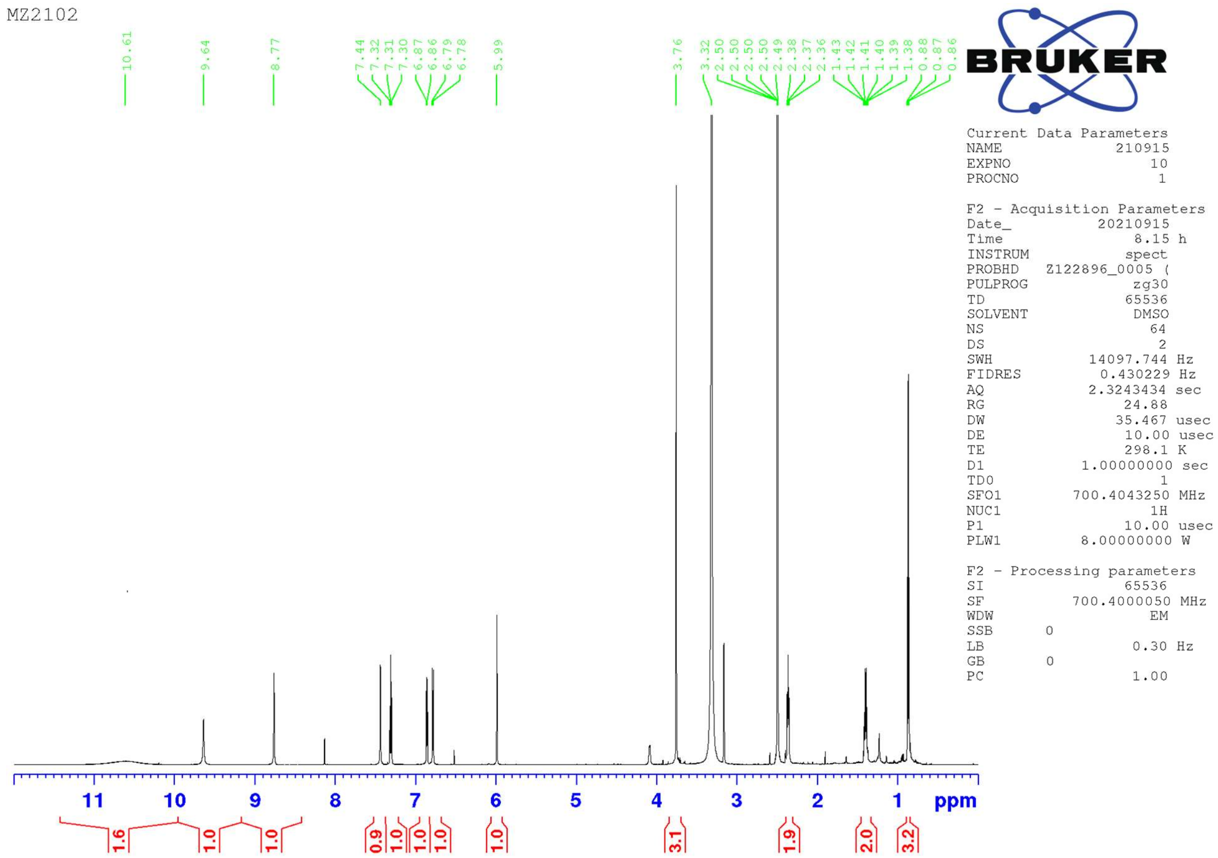

Figure S16. ^{1}H NMR spectrum of coprisidin A (**1**) in DMSO-d_6 at 700 MHz.

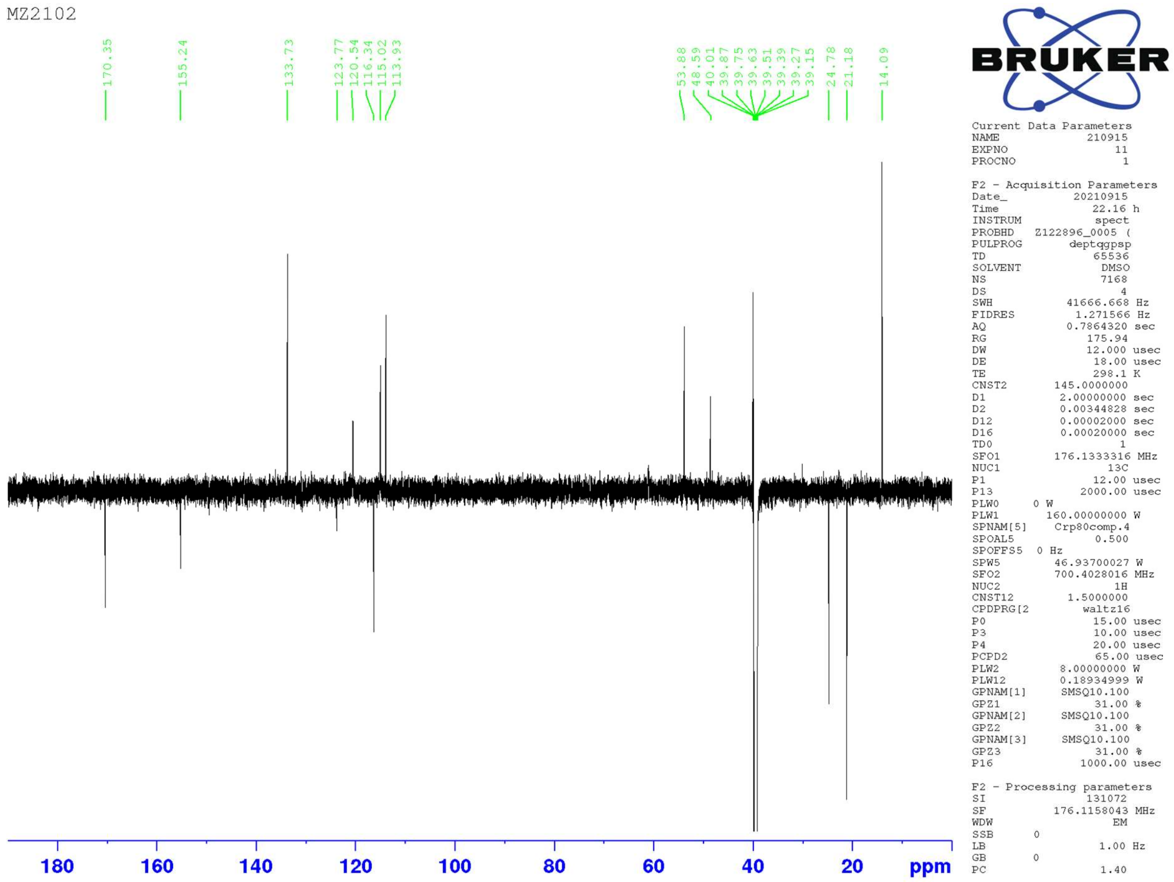

Figure S17. ^{13}C NMR spectrum of coprisidin A (**1**) in DMSO-d_6 at 176 MHz.

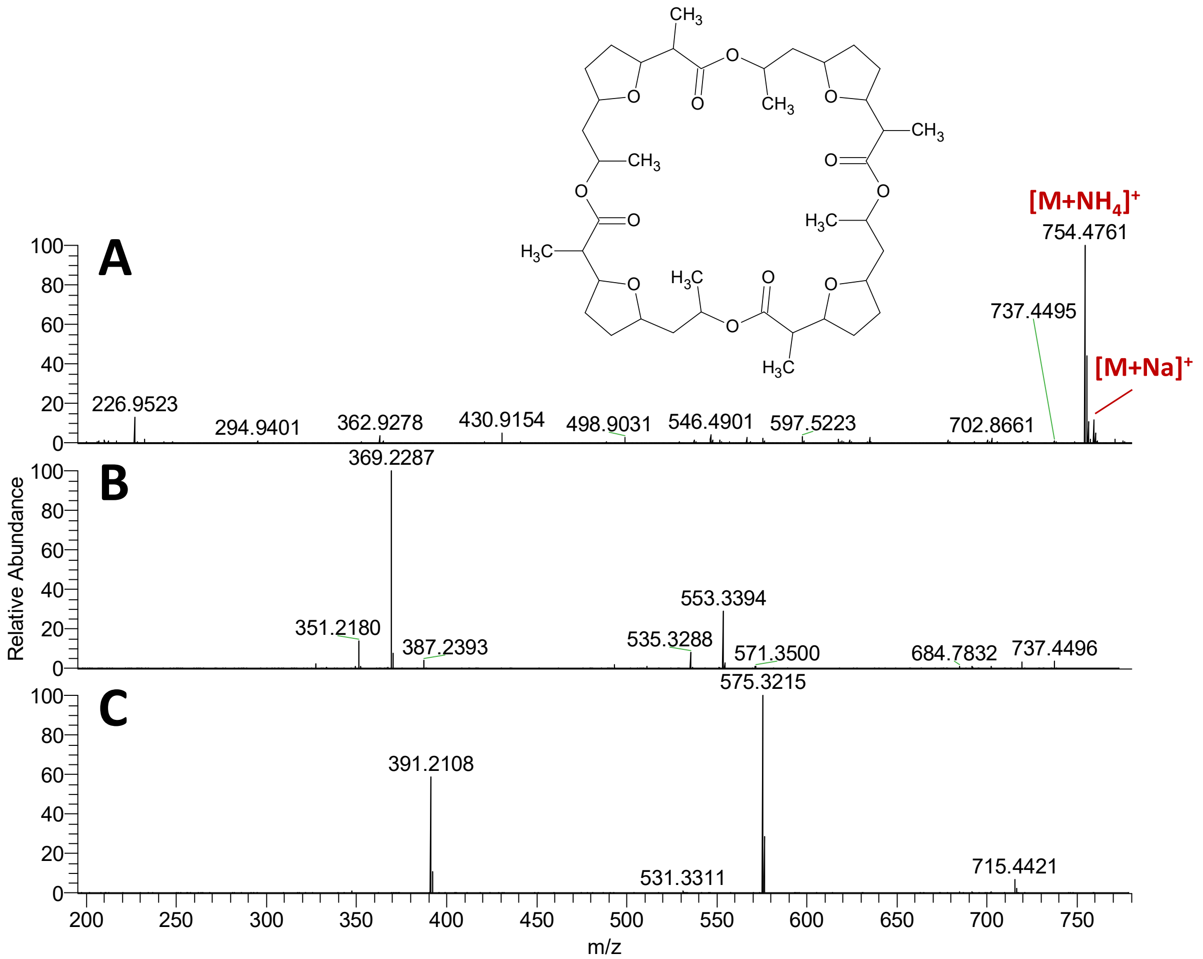

Figure S18. High resolution ESI mass spectrum of nonactin (**10**) at R_t 42.05-42.26 min (A), high resolution MS/MS spectrum of its $[M+NH_4]^+$ ion at *m/z* 754.4761 (B), and high resolution MS/MS spectrum of its $[M+Na]^+$ ion at *m/z* 759.4316 (B).

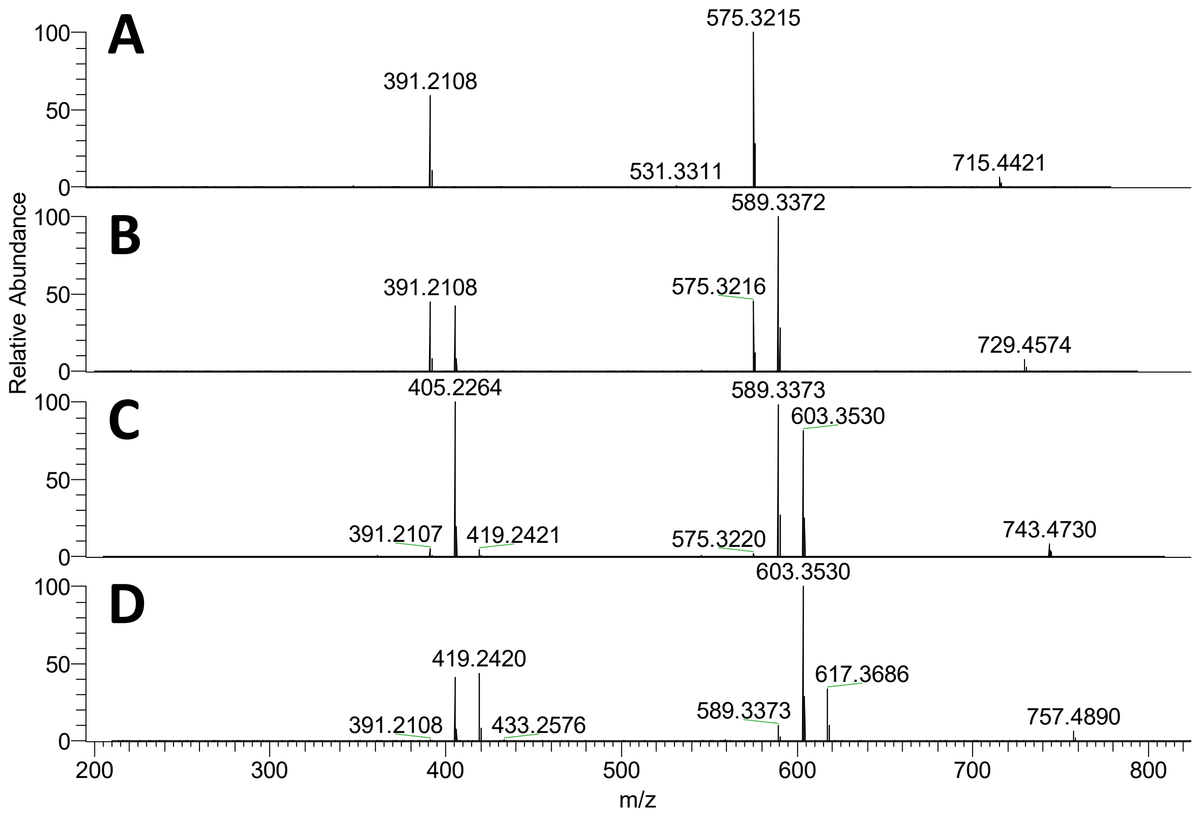

Figure S19. High resolution MS/MS spectra of the $[M+Na]^+$ ions of nonactin (**10**) at *m/z* 759.4316 (A), monactin (**11**) at *m/z* 773.4463 (B), isodinactin (**12**) at *m/z* 787.4616 (C), and either trinactin or isotrinactin (**13**) at *m/z* 801.4781 (D).

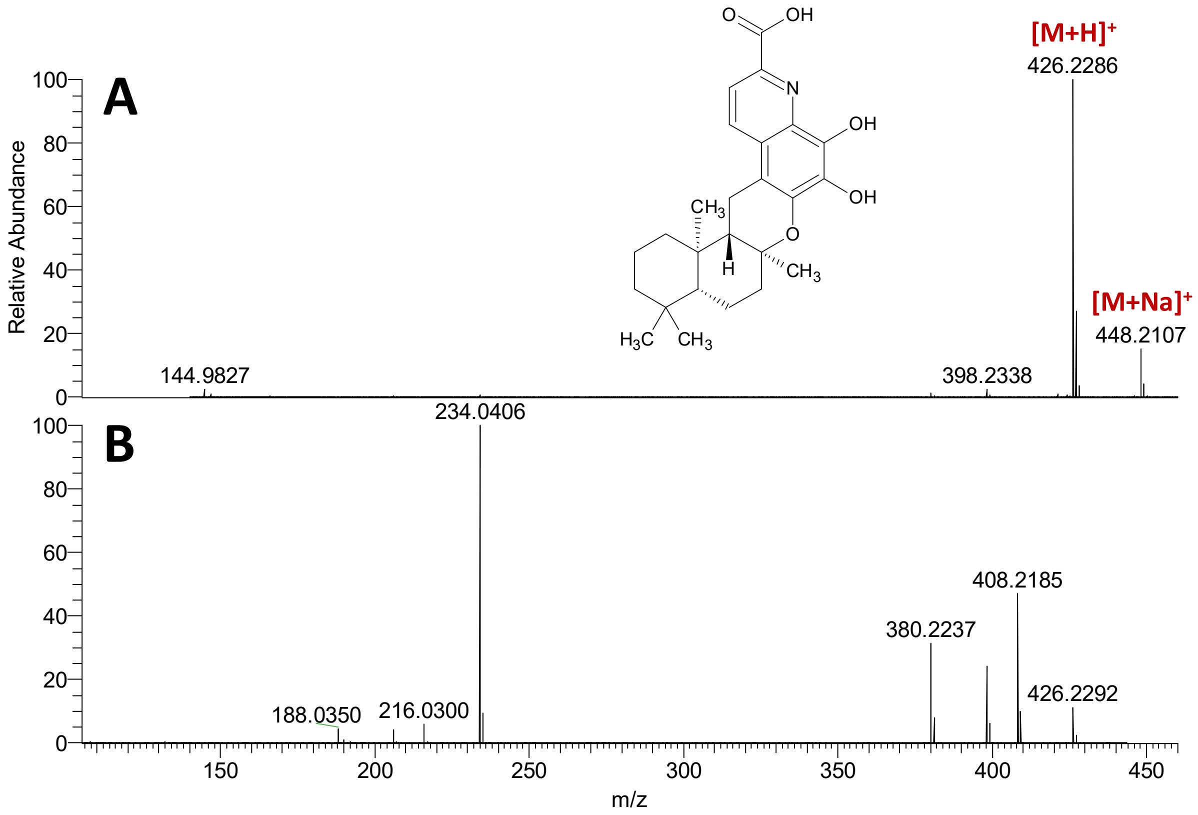

Figure S20. High resolution ESI mass spectrum of saccharoquinoline (**14**) at R_t 31.04-31.31 min (A) and high resolution MS/MS spectrum of its $[M+H]^+$ ion at *m/z* 426.2286 (B).

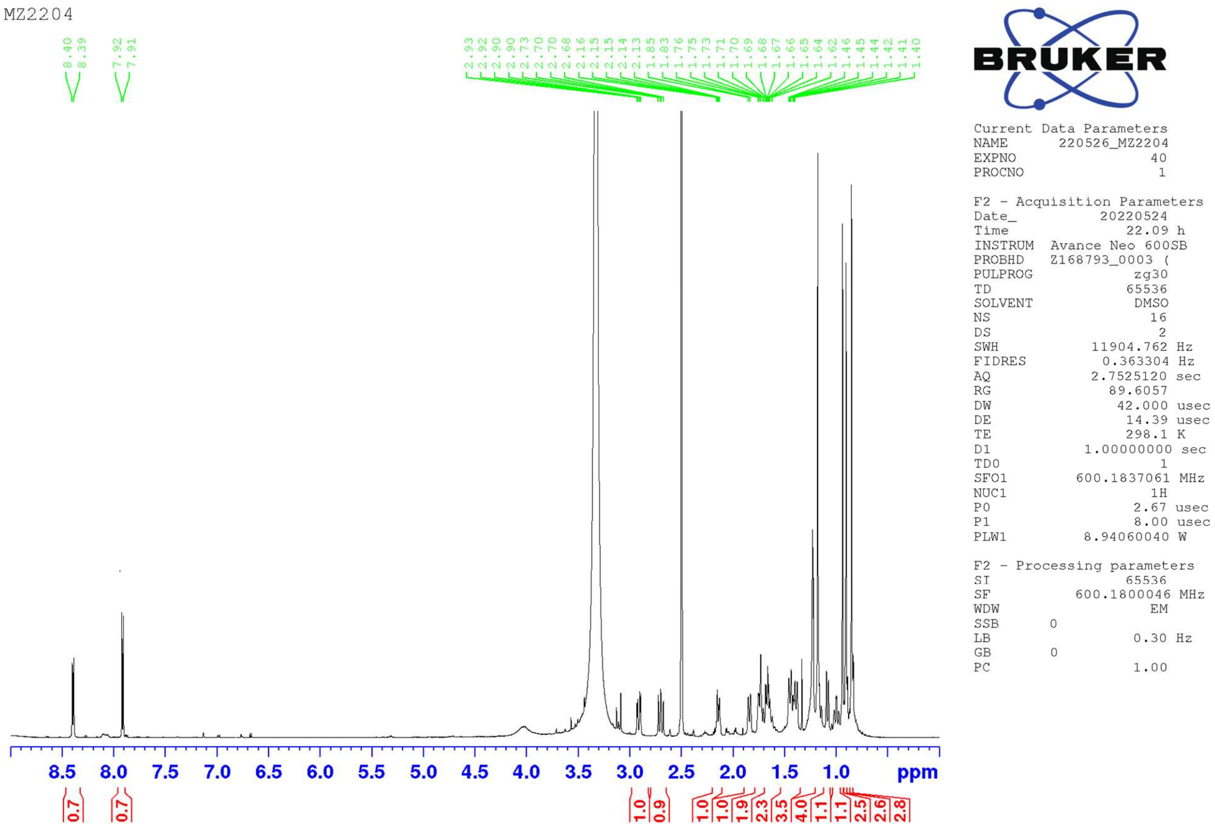

Figure S21. ^{1}H NMR spectrum of saccharoquinoline (**14**) in DMSO-d_6 at 600 MHz.

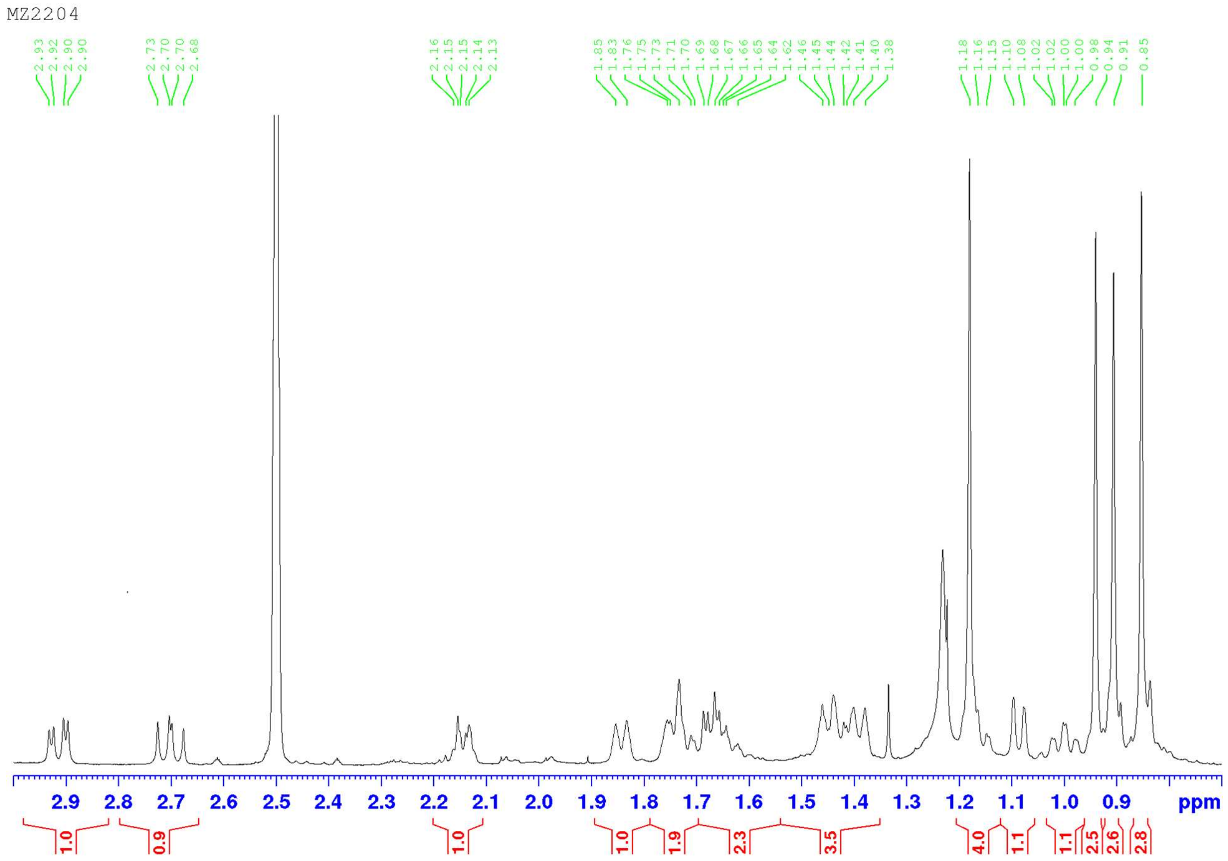

Figure S22. ^{1}H NMR spectrum of saccharoquinoline (**14**) in DMSO-d_6 at 600 MHz (zoom).

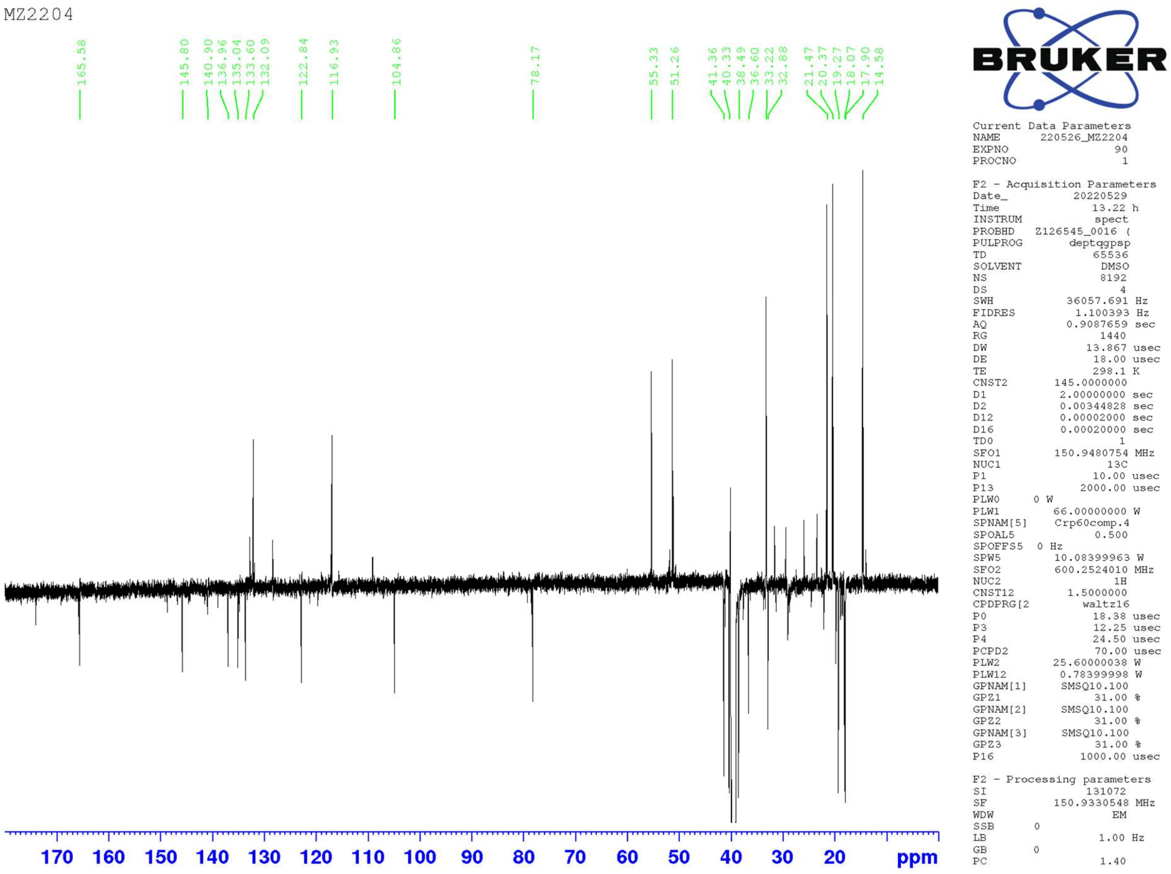

Figure S23. ^{13}C NMR spectrum of saccharoquinoline (**14**) in DMSO-d_6 at 151 MHz.

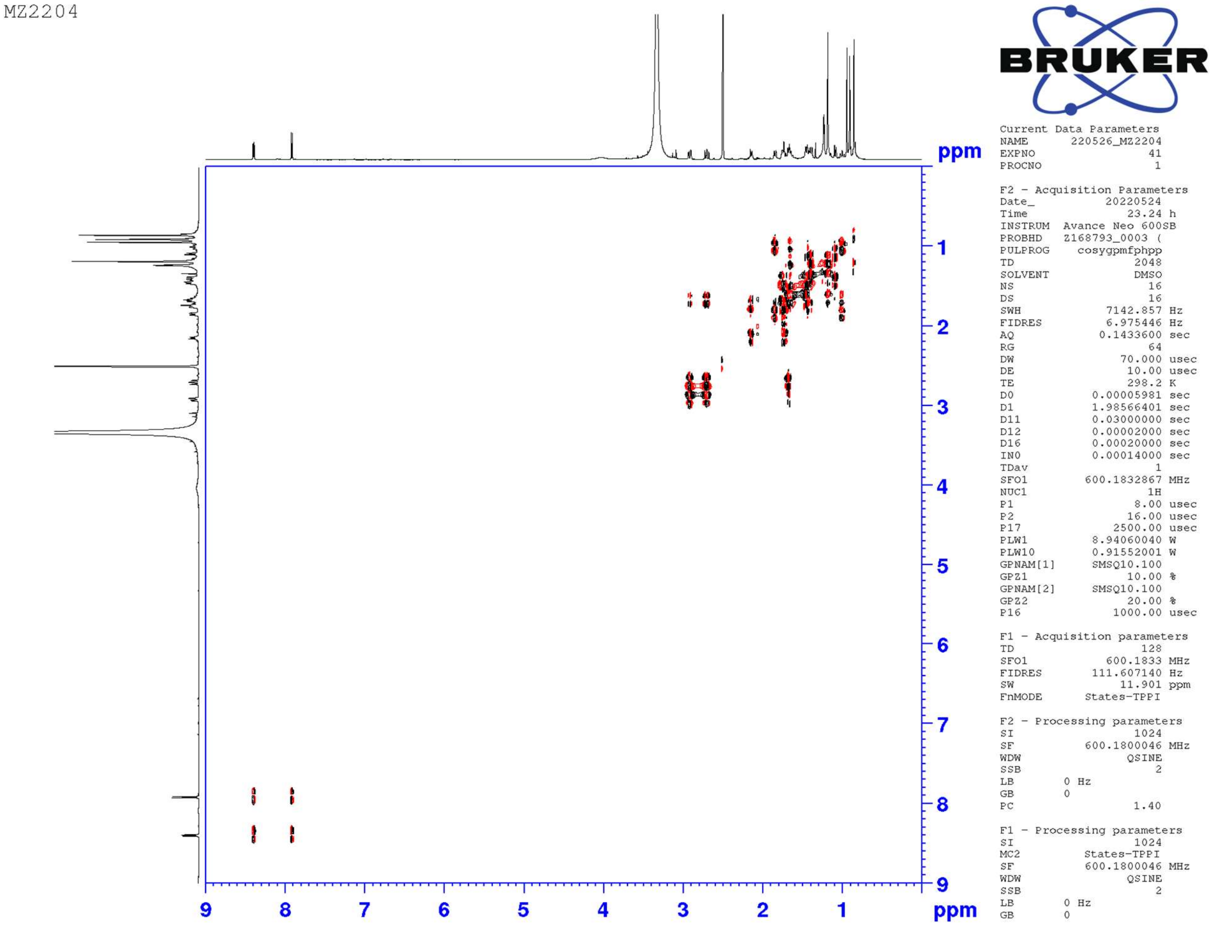

Figure S24. COSY spectrum of saccharoquinoline (**14**) in DMSO-d_6 at 600 MHz.

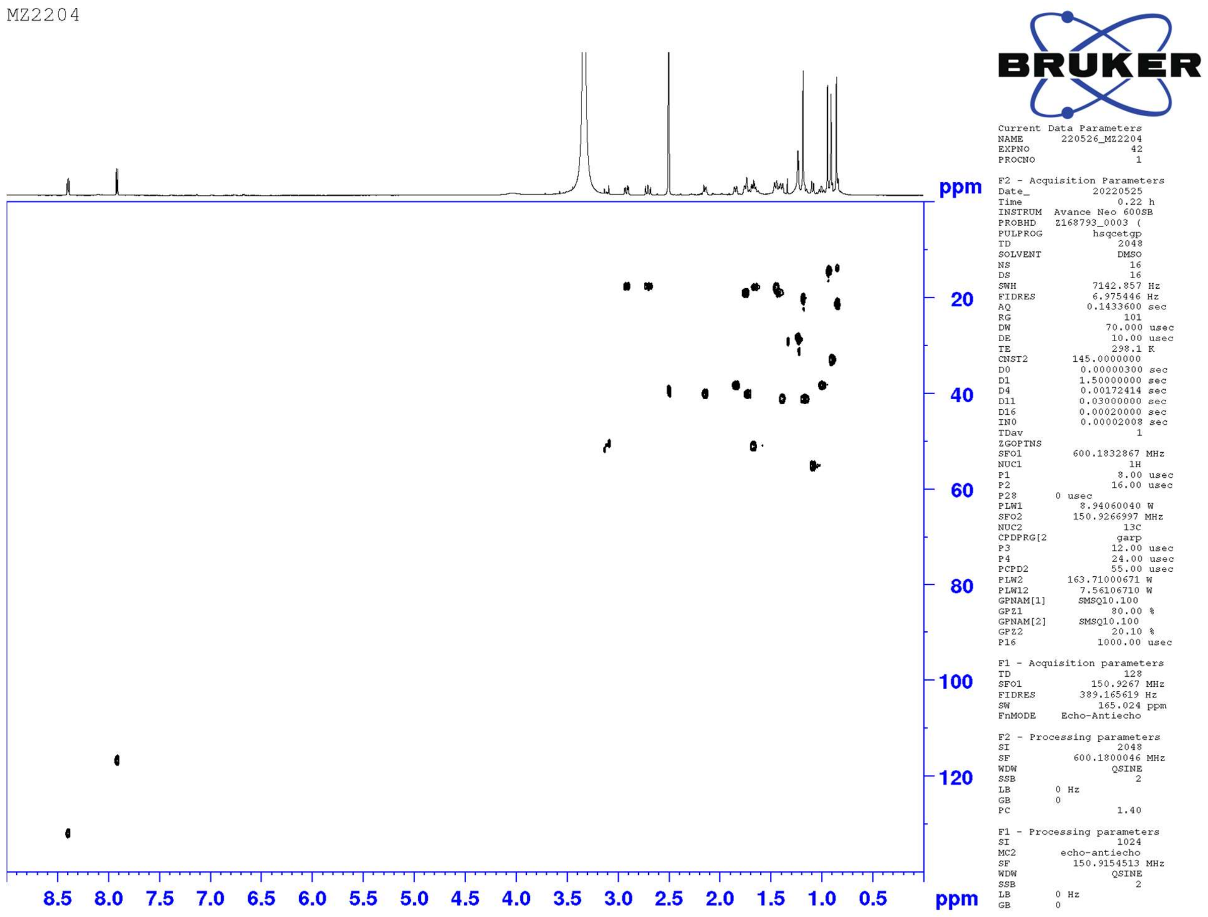

Figure S25. HSQC spectrum of saccharoquinoline (**14**) in DMSO-d_6 at 600 MHz.

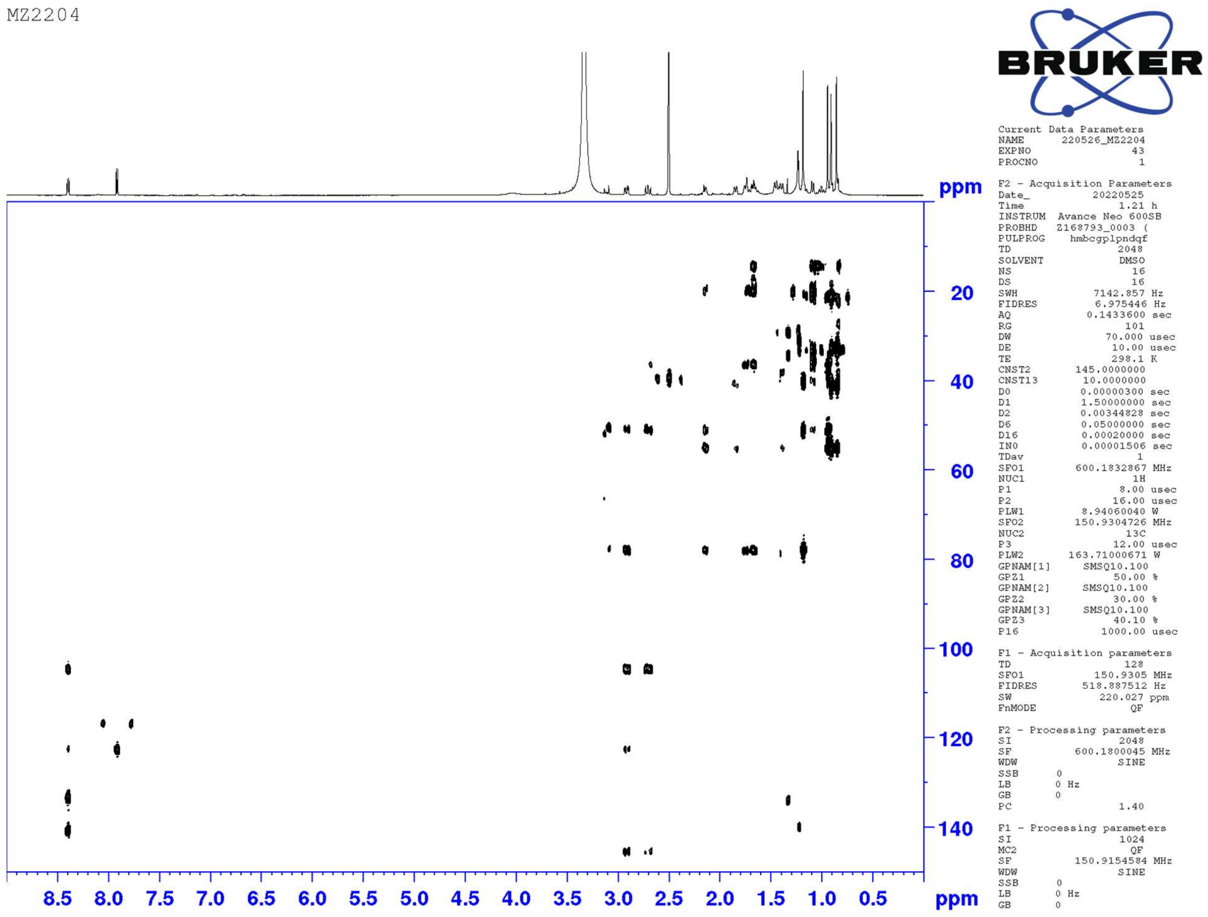

Figure S26. HMBC spectrum of saccharoquinoline (**14**) in DMSO-d_6 at 600 MHz.
